# Controversies and insights into cytokine regulation of neurogenesis and behavior in adult rodents

**DOI:** 10.3389/fimmu.2025.1550660

**Published:** 2025-04-25

**Authors:** Rodrigo Daniel Sepúlveda-Cuéllar, Diego Alberto Soria-Medina, Irma Cañedo-Solares, Fernando Gómez-Chávez, Liliana Monserrat Molina-López, María Yolanda Cruz-Martínez, Dolores Correa

**Affiliations:** ^1^ Programa de Doctorado en Ciencias Biomédicas, Universidad Nacional Autónoma de México (UNAM), Ciudad de México, Mexico; ^2^ Centro de Investigación en Ciencias de la Salud, Facultad de Ciencias de la Salud, Universidad Anáhuac México, Huixquilucan, EdoMex, Mexico; ^3^ Facultad de Psicología, Universidad Nacional Autónoma de México (UNAM), Ciudad de México, Mexico; ^4^ Laboratorio de Inmunología Experimental, Instituto Nacional de Pediatría (INP), Secretaría de Salud, Ciudad de México, Mexico; ^5^ Laboratorio de Enfermedades Osteoarticulares e Inmunológicas, Sección de Estudios de Posgrado e Investigación, Escuela Nacional de Medicina y Homeopatía (ENMyH), Instituto Politécnico Nacional (IPN), Ciudad de México, Mexico

**Keywords:** cytokine, hippocampus, immune system, neurogenesis, olfactory bulb, social behavior, subventricular zone, chemokine

## Abstract

Adult learning, memory, and social interaction partially depend on neurogenesis in two regions: the hippocampus and the subventricular zone. There is evidence that the immune system is important for these processes in pathological situations, but there is no review of its role in non-pathological or near-physiological conditions. Although further research is warranted in this area, some conclusions can be drawn. Intrusive LyC6hi monocytes and autoreactive CD4+ T cells have a positive impact on neurogenesis and behavior, but the latter are deleterious if specific to external antigens. Mildly activated microglia play a crucial role in promoting these processes, by eliminating apoptotic neuronal progenitors and producing low levels of interleukins, which increase if the cells are activated, leading to inhibition of neurogenesis. Chemokines are poorly studied, but progenitor cells and neurons express their receptors, which appear important for migration and maturation. The few works that jointly analyzed neurogenesis and behavior showed congruent effects of immune cells and cytokines. In conclusion, the immune system components -mostly local- seem of utmost importance for the control of behavior under non-pathological conditions.

## 1 Introduction

The nervous and immune systems connect us with the external environment and regulate internal biological functions to maintain homeostasis. Moreover, they interact in ways that were not even suspected half a century ago, even though conventional wisdom suggested such a connection for many years (1, 2). It is now well established that the immune system plays a crucial role in maintaining central nervous system (CNS) homeostasis, as well as in the onset or progression of various diseases (3–6).

Several reviews on these fascinating phenomena have suggested the role of specific immune cells, cytokines/chemokines, or related intracellular signaling on memory, learning and social behavior, as well as in neurogenesis in the subgranular zone (SGZ) of the hippocampus, the subventricular zone (SVZ) and the olfactory bulb (OB) of most vertebrates (7–13). Even though, most of the reviews have focused on diseases in which neuroinflammation is important and few have included the impact on non-pathological conditions. Additionally, there is considerable heterogeneity as reviews focus on multiple species, complicating comparisons. Moreover, the SVZ has received less attention in the context of behavior, so many reviews ignore this zone (5, 9, 14–18).

In the present review, we focus on *in vitro* studies of adult rodent cells or *in vivo* models without baseline disease. Studies on the effect of long-term cytokine treatment or chronic effects of acute exposure on the hippocampus, SVZ or OB, as well as those employing knockout (KO) models for cytokines or their receptors, were included. Models of neurodegenerative diseases, traumatic injuries, ischemia, stroke, infection or acute neuroinflammation were omitted, as well as those studies carried out in non-rodent species to minimize variability and improve the validity of conclusions regarding neurogenesis and cognitive aspects. On the other hand, we focus on animals whose brains have completed their development. Finally, we specifically review studies investigating cytokine receptor expression in neural stem cells, neuroblasts, and mature neurons within the hippocampus and the SVZ-olfactory bulb (SVZ-OB) systems.

To our knowledge, this review is the first to take a fully integrative approach to non-pathological interactions between the cytokines, the two neurogenic niches in adult rodents, and their possible outcome in memory and social behavior.

## 2 Memory and social behavior

Memory refers to the ability of the brain to encode, store, and later retrieve information. The hippocampus, a brain region in the medial temporal lobe, is involved in long-term memories, which are studied in paradigms such as Morris Water maze (spatial memory) and contextual fear conditioning (19–21). The hippocampus comprises the dentate gyrus (DG), the Cornu Ammonis structures -CA1, CA2, CA3, and CA4- and the subiculum. These regions are densely interconnected and communicate through intricate neural circuits and play a fundamental roles in memory retrieval, classified as declarative or explicit memory. For a comprehensive understanding of these circuits, see Lisman (22)Basu and Siegelbaum (23) and Eichenbaum (24).

On the other hand, social behavior refers to the ability of an individual to interact and communicate with other members of its species, maximizing its survival and reproductive success. These behaviors include aggression, mating, parental care, and communication (25–27). The relationship between social behavior and the olfactory system is well-known; because, during social interaction, brain structures associated with smell in mammals are activated, including the olfactory bulb, the piriform cortex, the nucleus of the lateral olfactory tract and the olfactory tubercle, among others (28). This circuit serves as an essential sensor that regulates physiological and behavioral responses by detecting small volatile and water-soluble molecules present in the environment, known as odorants (25, 29).

## 3 Neurogenesis in the hippocampus

**Figure 1:**
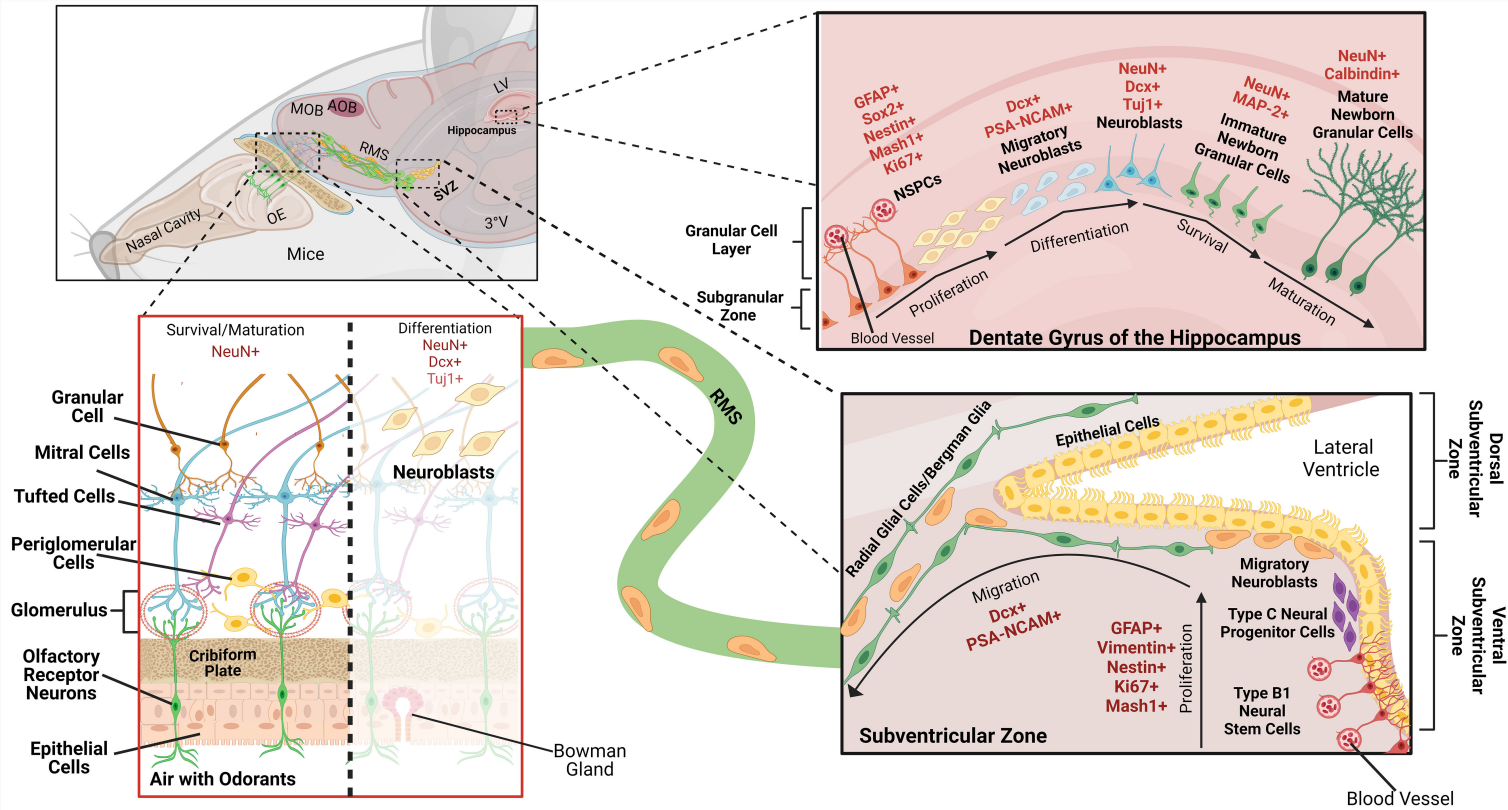
Representation of the neurogenesis in the hippocampus, the subventricular zone (SVZ) and the olfactory bulb (OB) of the mouse, along with the cell types that make up this phenomenon and their markers (in red). MOB, main olfactory bulb; AOB, accessory olfactory bulb; OE, olfactory epithelium; RMS, rostral migratory stream; LV, lateral ventricle; 3°V, third ventricle. The scheme was made in BioRender License *MJ27W1N9FE.*.

Neurogenesis in adult mammals was first described in the rat hippocampus many years ago, specifically in the SGZ of the DG (7). In **Figure 1**, we summarize the general process and specify the markers used in the works included in the present review. To deepen the understanding of the process, refer to several extraordinary papers (13, 30–33).

Newborn cells committed to the neural lineage originate from neural stem cells/neural progenitor cells (NSCs/NSPCs, hereinafter referred to as NSPC), which differentiate into intermediate progenitors and neuroblasts, stimulated by the neurotransmitter gamma-amino-butyric acid, GABAergic inputs. These cells migrate radially from SGZ to the granular layer. Subsequently, they form glutamatergic connections onto CA3 and become mature neurons with strong synaptic plasticity. The new cells will integrate into pre-existing neuronal circuits in the hippocampus (13, 32). Each cell exhibits distinct molecular markers depending on its developmental stage, frequently used for identification: NSPCs can be detected by Nestin, Ki67, Mash1 (Ascl1), Tbr2, and Sox2. Neuroblasts express doublecortin (DCX), polysialylated neuronal cell adhesion molecule (PSA-NCAM), and Tuj1; and mature neurons express Calbindin and NeuN (9, 34).

Neurogenesis in the DG participates in processes such as learning, memory and pattern separation, favoring the conversion of short-term memory into long-term memory, which can be retrieved when required, allowing the subject to incorporate new knowledge into the response repertoire (13, 33). Neurogenesis also promotes cognitive flexibility because it prevents interference between new and previously stored memories, allowing the development of novel strategies in response to a changing environment (30, 35).

It has been widely demonstrated that numerous factors can enhance neurogenesis, including an enriched environment, exercise, dietary restriction, estrogen, luteinizing hormone, pheromones, and, as will be discussed in this review, cytokines and immune cells (36–40). Several of these stimuli promote the production of different trophic factors such as brain-derived neurotrophic factor (BDNF), neurotrophin-3 (NT-3), nerve growth factor (NGF) or vascular endothelial growth factor (VEGF), which mediate the proliferation, differentiation and survival of the NSPCs; some of them are also involved in synaptogenesis (37, 41, 42).

Increased neurogenesis has been associated with better performance in spatial memory and pattern separation tasks (36, 40, 43–45). In contrast, reduced neurogenesis has been linked to impairment in contextual fear learning and spatial memory (46, 47). Nevertheless, there is some controversy, since some studies have reported that a decrease in neurogenesis did not influence hippocampal-dependent memory (48, 49).

It is important to mention that neurogenesis begins with an intensive proliferation of the undifferentiated cells, NSPCs, but most of them die because they lack sufficient response to signals that allow them to proceed to the next step. Only 40% become neuroblasts, and from these, a smaller percentage become mature neurons since there is a second wave of apoptosis (31).

## 4 Neurogenesis in the subventricular zone and the olfactory bulb

Bipolar neurons called olfactory sensory neurons (**Figure 1**) detect odors in the OB. These neurons form excitatory and inhibitory synapses with periglomerular, tufted, and mitral cells. This synaptic region, the glomerulus, is activated in response to specific odors (29, 50).

The first study of neurogenesis in the SVZ-OB was carried out by Doetsch, García-Verdugo, and Álvarez-Buylla in 1997. This region serves as the neurogenic niche of the OB in the murine adult brain. The SVZ contains type B1 and B2 cells, located below the lateral ventricles. The first are bipolar, with processes that directly contact the blood vessels of the blood-brain barrier (BBB) on one side and the cerebrospinal fluid (CSF) on the other (51, 52). B1 cells differentiate into type C cells and produce neuroblasts that express DCX and PSA-NCAM. They can further differentiate into different types of interneurons, astrocytes, or oligodendrocyte progenitor cells produced in different subregions of the SVZ (53–56).

The proliferation and migration of adult-born OB interneurons are tightly regulated by trophic factors, mainly the fibroblast growth factor 2 (FGF-2) (57) and epidermal growth factor (EGF) (58), as well as by GABA and glutamate through bidirectional communication between astrocytes and neuroblasts, providing robust homeostatic control over neurogenesis (59). Neuroblasts then migrate in chains through the rostral migratory stream (RMS) towards the OB, where those that survive differentiate into multiple subtypes of interneurons that integrate into local circuits (**Figure 1**) (11, 52, 60). The vast majority of neuroblasts that reach the OB differentiate into granule cells (94%), while some become periglomerular cells (4%) or astrocytes (<2%) (11, 61). All of these cell types play a crucial role in inhibiting and synchronizing glutamatergic mitral cell activity. However, only 50% of the newly differentiated granule cells survive (62, 63).

Although the exact role of SVZ neurogenesis in mammalian behavior is not fully understood, it plays an important role in detection, discrimination, and learning of novel odors. For example, newly generated neurons have been associated with the recognition of familiar odors and their discrimination from new ones (64). Inhibition of neurogenesis in the SVZ induces an increase in the odor detection threshold, as well as changes in behaviors such as mounting between males and females, aggression between males and recognition of new conspecifics (65, 66).

Neurogenesis has been documented in the adult human SVZ. The group led by Curtis et al. (67–69) described the presence of type A, B and C cells in the subependymal layer of normal brains in three separate studies, and found that they are important for neuroregeneration in Huntington’s disease. It remains to be elucidated what is the result of this process in normal human behavior.

## 5 The role of the immune system components on neurogenesis, memory, and social behavior

As mentioned, the interdependence of the immune and nervous systems has long been recognized (reviewed in 2, 4). However, the study of specific actions of the immune response on neurogenesis and its impact on memory/learning and social behavior began later (18, 70). In the following sections we present the main findings that, as we will see, are sometimes controversial.

### 5.1 Immune cells

#### 5.1.1 Microglia

Microglia, CNS resident macrophages, are classically activated by infection or injury, switching from a ramified to an amoeboid morphology (71). Neurons produce CX3CL1 (fractalkine) which binds to CX3CR1 on microglia, inhibiting their inflammatory activity (72), and is the quiescent microglia which promote neurogenesis in the DG by phagocytosing newly born apoptotic cells (73, 74). Neither intracerebroventricular (i.c.v.) administration of CX3CL1 nor genetic deletion of CX3CL1 in mice alters NSPC proliferation or differentiation (75, 76). In contrast, CX3CR1 knockout (KO) mice or those treated with CX3CR1 blocking antibodies exhibit reduced proliferation and differentiation of newborn cells in the DG (75–80). Importantly, hippocampal NSPC cells do not express CX3CR1 (**Table 1**), thus, it seems that these effects are mediated indirectly through microglial signaling.

**Table 1:** Effect of cytokines on neurogenesis of the hippocampus and the subventricular zone-olfactory bulb of mice in vivo and in vitro models and in multiple tasks dependent on neurogenesis in these regions.

Cytokine	Hippocampus										Subventricular zone & olfactory bulb														
	Learning and memory						Neurogenesis *in vivo*				Neurogenesis *in vitro*				Social behavior			Neurogenesis *in vivo*				Neurogenesis *in vitro*			
	MWM	BMT	YM	CFC	PA	NOR	P	D	M	S	P	D	M	S	Exp	Agr	Soc	P	D	M	S	P	D	M	S
**IL-1β**	**↑**			**↑**																		**↑**			
							**=**	**=**							**==**										
	**↓↓**			**↓↓**			**↓↓↓**	**↓↓**			**↓**	**↓**			**↓**	**↓**		**↓**					**↓**		
**TNF-α**		**↑**				**↑**	**↑**	**↑↑**	**↑↑**	**↑↑**						**↑**		**↑**				**↑**			
	**=**						**=**											**=**							
							**↓↓**	**↓↓**	**↓↓**	**↓↓**	**↓**											**↓↓**			
**IL-6**	**↑↑↑**					**↑↑**	**↑**			**↑**								**↑↑**			**↑**				
				**=**			**=**	**=**																	
	**↓**	**↓**	**↓**	**↓**	**↓**		**↓**	**↓**		**↓**		**↓↓↓**					**↓**								
**IL-15**			**↑**	**↑**														**↑**	**↑**						
**IFN-α**	**↑**																								
										**=**		**=**											**=**		
							**↓↓**	**↓**		**↓**	**↓**						**↓**	**↓**				**↓**			
**IFN-β**	**↑**																								
																						**↓**	**↓**		
**IFN-γ**	**↑**						**↑**	**↑↑**									**↑**						**↑↑↑**		
	**↓**					**↓**	**↓**	**↓↓**	**↓**									**↓↓**	**↓**		**↓**	**↓↓**	**↓↓↓↓**		
**IL-4**	**↑↑↑**			**↑↑**																					
	**=**				**=**																				
											**↓**				**↓**										
**IL-17**								**↑**																	
	**=**		**=**	**=**													**=**								
							**↓**	**↓**	**↓**	**↓**															
**IL-23**								**↑**									**↑**								
				**=**																					
**IL-2**	**↑**																								
				**↓**				**↓**																	
**IL-10**	**↑**																	**↑**	**↑**	**↑**					
							**=**																		
	**↓**							**↓**		**↓**								**↓**	**↓**		**↓**				
**TGF-β**	**↑**	**↑**								**↑**							**↑**		**↑**						
								**=**	**=**										**=**	**=**		**=**			
							**↓↓↓**	**↓↓**	**↓↓**	**↓↓**								**↓**			**↓**	**↓**			
	**↑↑**			**↑**			**↑**					**↑**	**↑**					**↑**					**↑**	**↑**	
**GM-CSF**	**=**							**=**																	

P, proliferation; D, differentiation; M, maturation and S, survival. The learning and memory tests found were the MWM, Morris water maze; BMT, Barnes maze test; YM, Y maze; CFC, context-conditioned fear; PA, Passive avoidance; NOR, novel object recognition. Tests of social behavior are Exp, social exploration; Agr, aggressiveness test and Soc, sociability test. Each arrow indicates an item that found whether the effect was beneficial (green up arrow, red down arrow, blue equal sign); question marks indicate that no evidence of the cells that produce it has been found in the CNS. Each arrow represents the result of one study.

Behavioral studies in CX3CR1 knockout mice reveal controversial results. Some reports indicate deficits in spatial memory and fear conditioning (76, 79), with CX3CL1 levels in the hippocampus increasing after spatial learning tasks (81). In contrast, other works demonstrate improved cognitive performance in CX3CR1 KO mice (77, 78).

On the other hand, SVZ-OB microglia exhibit a unique phenotype characterized by reduced branching and some phagocytic activity (82). Ablation of microglia in the SVZ does not affect NSPC proliferation (83), and CX3CR1 KO mice display intact SVZ-OB neurogenesis despite enhanced olfactory memory (78). However, they support neuroblast migration through CX3CR1-independent mechanisms, by secreting insulin-like growth factor-1 (IGF-1) and cytokines such as IL-4 and IL-10, which promote neuroblast survival and integration into the olfactory bulb (84, 85).

**Figure 2:**
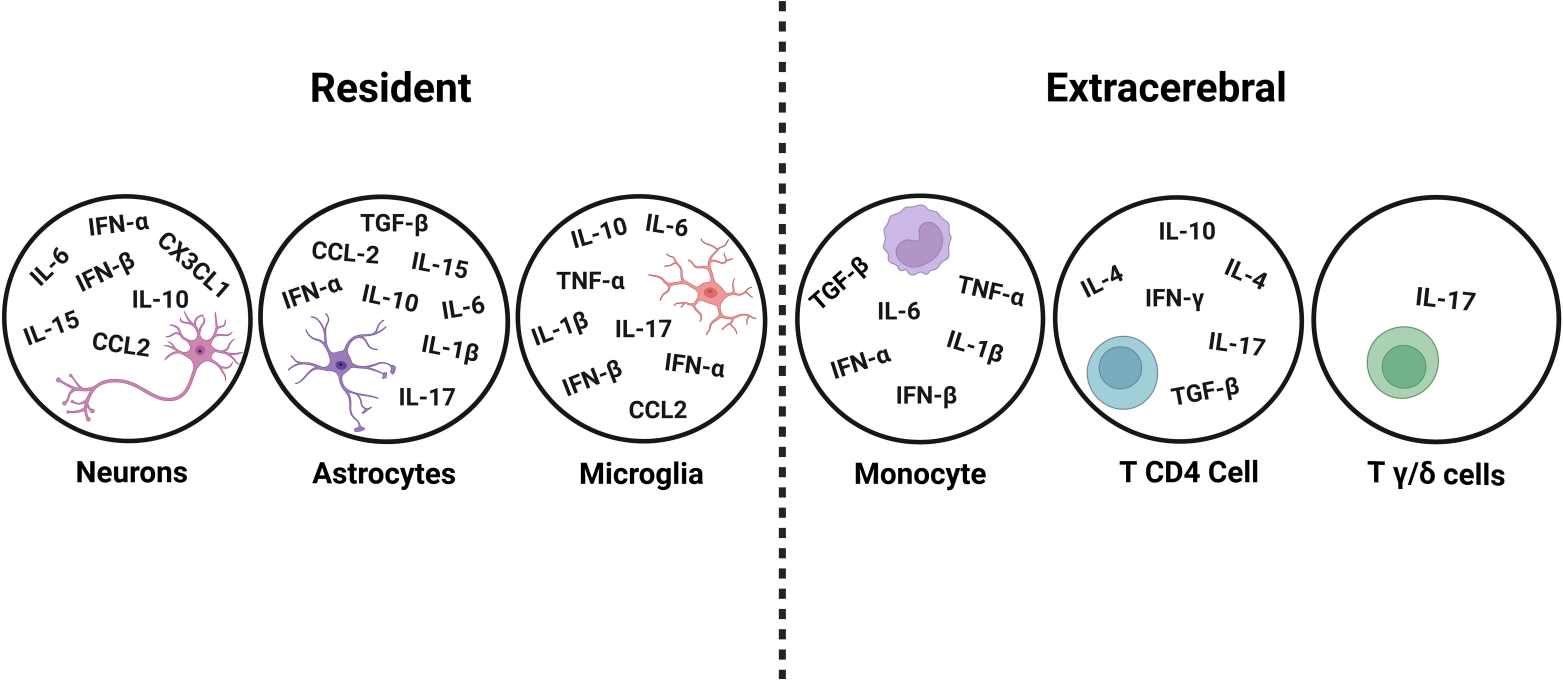
Representation of the CNS resident and extracerebral cells that produce cytokines or chemokines described in the text. The scheme was made in BioRender license *FA26YDR5NL*.

Activation of microglia enhances their phagocytic activity and the secretion of proinflammatory cytokines such as TNF-α, IL-1β and IL-6 (**Figure 2**) (71), which may negatively affect neurogenesis and probably also cognitive functions, as discussed below.

#### 5.1.2 Monocytes Ly6Chi

Ly6Chi monocytes, although not resident in the CNS, influence hippocampal neurogenesis when they arrive via CCL2/CCR2 signaling. Neurons and astrocytes produce CCL2, recruiting monocytes to the hippocampus, as demonstrated by reporter gene screening (86). Systemic depletion of Ly6Chi monocytes (via antibody blockade or CCR2 KO) reduces DG neurogenesis. These cells enhance neurospheres formation *in vitro*, via unidentified soluble factors. Potential mediators include IL-6 and TNF-α, which promote neurogenesis if in controlled amounts (87). NSPCs in the hippocampus express CCR2 (**Table 2**), so they may respond directly to CCL2. To our knowledge, there are no reports on the role of Ly6Chi monocytes in neurogenesis in the SVZ or social behavior.

**Table 2:** Chemokine and cytokine receptors found in different cellular phenotypes of the hippocampus and the subventricular zone.

Cytokine	Subgranular zone receptors			Subventricular zone receptors		
	NSPC	Neuroblast	Mature neuron	NSPC	Neuroblast	Mature neuron
IL-1β	R1	R1	R1	R1		
TNF-α	R1 R2			R1 R2		
IL-6	Rα		?	Rα		?
IL-15	?			IL-15Rα		IL-15Rα
INF-α & IFN-β	R1 R2		R1 R2			
IFN-γ	?			R1 R2		
IL-4	N.F.		Rα			
IL-17	R					
IL-23						
IL-2			Rα Rβ			Rα Rβ
IL-10	N.F.		N.F.	R1		
TGF-β	R1* R2 R3*		R2	R2		
GM-CSF	Rα	Rα	Rα	Rα		
CX3CL1	N.F.		N.F.	CX3CR1	N.F.	
CCL2	CCR2		CCR2	CCR2	?	CCR2
CCL3	CCR1 CCR5		CCR1 CCR5	CCR1 CCR2	?	CCR1 CCR5
CCL11	?	?	CCR3			
CXCL12	CXCR4	CXCR4	CXCR4	CXCR4		CXCR4

NSPC, Neural progenitor cell; N.F., Not found.?, suggested by functional studies, but not demonstrated directly.

#### 5.1.3 T cells

B and T lymphocytes mediate adaptive immunity (88). While B cells are dispensable, CD4+ T cells critically regulate hippocampal neurogenesis and behavior (89–92).

Immunodeficient mice (nude, SCID and RAG1/2 KO) display reduced DG cell proliferation and maturation, which is reversed by CD4+ T cell reconstitution (90, 91, 93). TCR specificity is important: MBP-specific T cells enhance DG neurogenesis, whereas ovalbumin-specific T cells depress it (92, 94). These effects are likely mediated by BDNF rather than by cell contact mechanisms (91). Furthermore, regulatory T cells stimulate NSPC proliferation via IL-10 in the SVZ (95).

Regarding cognitive aspects, CD4+ T cells specific for myelin basic protein (MBP)-or myelin oligodendrocyte glycoprotein (MOG) stimulate performance in the MWM (92, 96), whereas SCID, nude or anti-CD4-treated mice have deficits, reversed by T cell reconstitution (89, 97). RAG1 KO mice do not show impaired spatial memory, although deficient meningeal T cell trafficking correlates with cognitive decline (98, 99).

Only these three cell types have been reported to influence neurogenesis or cognitive functions, and as it will be seen, all mechanisms, except for the abovementioned scavenging activity of quiescent microglia, involve cytokines or chemokines.

### 5.2 Innate response cytokines and chemokines

Cytokines and chemokines are short peptides produced by various types of cells. They mediate the generation, proliferation/differentiation, activation, modulation, or migration of various cell types. They were initially called interleukins (ILs) since some are secreted by leukocytes and act upon other leukocytes (100). Today, they are called cytokines because the functions of all systems, including the endocrine and CNS, are highly dependent on them.

Several cytokines may indirectly affect the CNS through the “neural pathway” by activating sensory afferent fibers of the autonomic nervous system, such as the Vagus nerve, thus relaying information to the brain (1). Additionally, cytokines may be transported across the BBB through active mechanisms or are produced by BBB endothelial cells or leukocytes that invade the CNS, potentially crossing this narrow structure under pathological and possibly even non-pathological conditions (101, 102).

Leukocytes and cytokines may also come from a structure described in the 18th century by Paolo Mascagni but reliably demonstrated until recently, that is, the meningeal lymphatic network in the dura mater, which is formed postnatally (revised by 103). Furthermore, recent research has identified a mesothelial-like meningeal layer that compartmentalizes the subarachnoid space in the brain, called the subarachnoid lymphatic-like membrane, or SLYM (104). This structure is morpho- and immunophenotypically similar to the mesothelial membranes that line peripheral organs and body cavities. It consists of bone marrow-derived myeloid cells (macrophages and dendritic cells) that control the exchange of molecules larger than 3kDa. No doubt, its role in the transmission of immune molecules between colony-stimulating factor (CSF) and the parenchyma of the brain (and spinal cord) will soon be studied.

Finally, it has been shown that cytokines and chemokines can be produced within the CNS, by glial and even neuronal cells (105, 106; **Figure 2**).

#### 5.2.1 Interleukin 1 beta

Interleukin-1β (IL-1β) is an agonist of the IL-1 family, produced mainly by hematopoietic cells such as blood monocytes, tissue macrophages, skin dendritic cells and brain microglia and astrocytes, as well as endothelial cells in response to molecular patterns associated with pathogens (PAMPs), activated complement components and cytokines like TNF-α and IL-1 itself (107; **Figure 2**). In addition, IL-1β orchestrates the differentiation and function of innate and adaptive lymphoid cells, induces the production of other cytokines, and is also involved in acute phase protein synthesis by the liver, the induction of fever through the hypothalamus, and sickness behavior (108, 109).

In the CNS, the IL-1β type 1 receptor (IL-1R1) is expressed in the hippocampus by the pyramid cells of CA3 and CA4 regions (110, 111). The cytokine modulates the excitability and neurotransmission of neurons (106). Regarding the role of IL-1β in neurogenesis, it has been shown that both newborn cells and neuroblasts also express IL-1βR1 in mice and rats (112, 113; **Tables 1**, **3**). Intracerebroventricular or subcutaneous administration of IL-1β reduces proliferation and differentiation of NSPC in the DG. This effect is prevented by IL-1ra, the antagonist of IL-1R (112, 114). Moreover, in transgenic mice with chronic overexpression of IL-1β in the brain, DCX and NeuN cells are reduced, while the number of GFAP cells increases (115). Interestingly, IL-1βR1 KO does not affect the differentiation and survival of NSPC (112, 114). Seguin et al. (116) reported an increase in BrdU cells after four consecutive daily administrations of IL-1β via the intrahippocampal route, although they did not identify the phenotype of the proliferating cells. Notably, a single administration of IL-1β did not change the number of BrdU cells, whether injected locally or intraperitoneally.

**Table 3:** Effect of chemokines on learning and memory in mice and neurogenesis in the hippocampus and subventricular zone.

Chemokine	Original name	Learning and memory impact	*In vivo* hippocampal neurogenesis	*In vitro* subventricular zone neurogenesis
CCL2	MCP-1			Differentiation ↓ Migration ↑
CCL3	MIP-1α	PA↓		Differentiation ↑
CCL11	Eotaxin	YM↓ CFC ↓	Differentiation ↓	
CXCL1	GROα			Differentiation ↑
CXCL12	SDF1	MWM ↑	Differentiation ↑ Maturation ↓	
CX3CL1	Fractalkine		Proliferation = Differentiation =	

A positive effect is represented with an up arrow, a negative effect with a down arrow.

Studies performed *in vitro* supported these findings since the addition of IL-1β to adult hippocampal cells decreases the number of proliferating (BrdU+) cells (112), which is consistent with the results obtained by Ryan et al. (113), who reported a decrease in proliferation and differentiation to DCX in a dose- and time-dependent manner.

Regarding neurogenesis in the SVZ-OB axis, a single administration of IL-1β in the lateral ventricles decreases the number of proliferating cells in the SVZ. In a neurosphere culture obtained from this region, the cytokine directly increases the number of the NSPCs and reduces their differentiation into MASH1 cells, thus inhibiting lineage progression (117). In particular, NSPCs display IL-1R1, and the cytokine could be produced by microglia or astrocytes, or it could arrive from external sources (**Table 2**).

The involvement of IL-1β in memory and learning seems paradoxical. For example, overexpression in the hippocampus has been associated with impaired spatial memory in MWM (118), further confirmed by Hein et al. (119), who found conditioning deficits in spatial memory and contextual fear using the same transgenic model. In contrast, experiments with IL-1R1 KO mice have shown deficiencies in these tasks (120). Thus, physiological levels of IL-1β are likely necessary to maintain proper memory and learning.

As in the DG, SVZ NSPCs express the IL-1β receptor 1 (**Table 3**), but the origin of the cytokine could be local (microglia or astrocyte) or external (**Figure 2**) since it is long known that it reaches the brain.

There are contradictory results regarding SVZ-OB-related behavior. Chronic subcutaneous administration of IL-1β for four weeks impairs social exploration of the mice, but IL-1R KO mice show no change in this behavior, while aggressive behavior increases in interleukin gene knockout mice (114, 121, 122).

In summary, IL-1β in the SGZ and SVZ mainly leads to reduced NSPC proliferation and differentiation. Furthermore, both overproduction and inhibition of IL-1β can harm contextual and spatial memory. This cytokine also affects social behavior, but more studies are needed to clarify this.

#### 5.2.2 Tumor necrosis factor alpha

Tumor Necrosis Factor-α (TNF-α) coordinates the inflammatory immune response and exhibits pleiotropic effects on various cell types (123). For example, it activates neutrophils, causes muscle catabolism and, like IL1-β, induces acute phase proteins and fever (88). TNF-α is initially produced as an active transmembrane protein, serving as a precursor to the soluble form generated through processing by the TNF-α-converting enzyme (124). Both forms of TNF-α exhibit a binding preference for the TNF-α receptor 1 (TNFR1).

This interaction leads to inflammation by triggering the production of other inflammatory cytokines and chemokines, as well as cell death by apoptosis. When TNF-α interacts with the type 2 receptor (TNFR2), it may also induce apoptosis, but it can protect the cell from death as well (88, 125, 126).

Inflammatory stimuli in the CNS induce the production of TNF-α by microglia, neurons, astrocytes, and infiltrating immune cells, specifically monocytes (**Figure 2**). Furthermore, soluble TNF-α crosses the BBB through a specific saturable transport system to the brain under physiological conditions. It has different effects depending on the type of receptor and the form of cytokine, such as inducing the death of dopaminergic neurons or protecting the myelin sheath (127). While TNFR1 is related to inflammation and glutamatergic and dopaminergic activity, TNFR2 downregulates it in the periphery and has a neuroprotective and repairing effect in the CNS (127).

Regarding neurogenesis, the progenitor cells (Nestin) of the hippocampus express TNF-α and its receptors (128; **Table 1**). Seguin et al. (116) reported a decrease in BrdU cells in the DG 24 h after a single i.p. injection (1μg) of the cytokine. However, this effect is probably indirect, since no significant difference in proliferation were identified when TNF-α was administered directly to the hippocampus (0.05 μg), either 24 h or five days prior. In contrast, Chen and Palmer (129) reported that infusion of TNF-α directly into the DG reduces the differentiation, maturation, and survival of newly generated neurons, an observation supported by the opposite effect in TNFR1 or TNF-α KO mice. The same observation was reported by Iosif et al. (128) using a TNFR1 KO model. Interestingly, TNFR2-deficient mice presented the opposite effect (129), consistent with its neuroprotective role (125–127). In agreement, Matsuda et al. (130) demonstrated inhibition of NSPC replication *in vitro* in a dose-dependent manner in the range between 10 and 50 ng of the cytokine.

Regarding the effect of TNF-α in the SVZ (**Table 2**), it has been shown that i.c.v. administration increased the number of proliferating cells in the SVZ; however, the identity of these cells was not explicitly specified (131). It was shown that TNF-α receptor-1 is expressed by NSPCs in this area (**Table 1**). Still, deletion of the receptor or cytokine did not affect the basal number of BrdU or Proliferating Cell Nuclear Antigen positive (PCNA) cells. However, the effect of the other receptor still needs to be studied (132). *In vitro*, 10ng/mL of TNF-α inhibited proliferation and decreased the percentage of β-tubulin III cells (an early neuron marker) in SVZ-derived NSPC cultures (133).

More recently, Belenguer et al. (134) demonstrated that low dose (0.1 ng/ml) of murine TNF-α increases the proliferation of subependymal zone NSPCs, but a higher concentration (10 ng/mL) inhibits it. In the same study, TNF-α was shown to activate both receptors, with opposite effects: TNFR2 moves NSPCs to activated states, while TNFR1 promotes their return to the “deep quiescence” and differentiation multipotentiality; this mechanism ensures that changes in basal levels in the microenvironment will not exhaust the NSPC pool (134). In apparent contradiction, Widera et al. (135) reported an increase in the number of BrdU cells in NSPC cultures after the addition of 4.0 and 10.0 ng/mL of TNF-α, although, they used human TNF-α, which precludes any conclusive interpretation (136).

In summary, most studies have shown that high levels of TNF-α reduce the proliferation, differentiation, maturation, or survival of NSPC in both the DG and SVZ. However, it is suggested that it has opposite effects depending on the receptor to which it binds *in vivo*.

Regarding cognitive functions, Scherbel et al. (137) reported no significant differences between TNF-α KO and wild-type C57BL/6 mice in spatial learning using the MWM test. More recent studies suggest the opposite, as TNFR1, TNFR2, and TNF-α KO mice of the same genetic background show deficits in hippocampus-dependent memory, assessed in the Barnes spatial maze and the novel object recognition test (138, 139).

Much less information is available on TNF-α and social behavior: the only report showed ablation of aggressive conduct in TNFR1 and TNFR2 KO mice (140).

*In vivo* and *in vitro* studies of the effect of TNF-α on neurogenesis must carefully evaluate the role of the two receptors and different concentrations of the cytokine, along with simultaneous changes in the levels of other cytokines.

#### 5.2.3 Interleukin 6

IL-6 is a proinflammatory cytokine initially called “B-cell stimulating factor-2” due to its ability to induce immunoglobulin production, and cloned in 1986 (141). IL-6 exerts its proinflammatory functions by binding to the IL-6 receptor, IL-6R, and this binary complex can interact with gp130, a ubiquitously expressed receptor (142).Although only those cells that express IL-6R are traditionally considered to be affected by IL-6 (143, 144), the metalloproteinase ADAM17 can release IL-6R from the membrane (145). Soluble IL-6R (sIL-6R) can bind IL-6 and subsequently interact with gp130, even in cells that do not express IL-6R. This process is called IL-6 trans-signaling (146–148). Through these mechanisms, IL-6 acts as a primary regulator of immune cell metabolism, proliferation, and differentiation.

This cytokine is highly expressed in the central nervous system (149), with astrocytes being the primary source. However, it can be synthesized by microglial and neural cells and can come from infiltrating monocytes (**Figure 2**). Its production is regulated by IL-1β, TNF-α, neuropeptides, and neurotransmitters such as norepinephrine and serotonin. At the cellular level, IL-6 has an inhibitory effect on sodium (Na^+^) and calcium (Ca^2+^), which may serve as neuroprotective mechanisms in the CNS (106). *In vitro* studies have reported mRNA expression of its receptor in NSPC obtained from the SGZ and SVZ (**Table 2**) (150, 151).

Regarding its role in neurogenesis, it has been shown that exposure of NSPCs derived from the hippocampus to media conditioned by IL-6 decreases their differentiation into neurons but not into oligodendrocytes or astrocytes, the latter being even increased (152, 153). Furthermore, Oh et al. (154), observed an increase in Tuj1 cells in NSPC cultures after administering anti-IL-6 antibodies. *In vivo*, these observations are supported by studies showing that transgenic mice overexpressing IL-6 in the brain exhibit decreased proliferation, differentiation, and survival of newly formed neural cells in the SGZ (155). Paradoxically, Bowen et al. (156) studied both neurogenic regions in IL-6 KO mice and observed decreased proliferation and survival of NSPCs in the DG and the SVZ; and Storer et al. (151) demonstrated that IL-6 is necessary for NSPC self-renewal and proliferation in the SVZ. However, Seguin et al. (116) obtained apparently different results, i.e. they observed no changes in the proliferation or number of DCX cells in the DG of mice with acute intraperitoneal or intrahippocampal administration of IL-6, indicating a lack of differentiation into neurons.

Current evidence points to conflicting effects of IL-6 on behavior. IL-6 KO mice showed improved performance in the radial maze (157); but using the same model, other researchers later found deficits in novel object recognition and MWM tasks (158–160). On the other hand, mice overexpression of IL-6 from birth presents deficits in passive avoidance and spatial memory (161, 162). Consistently, transgenic GFAP-IL6 mice show impairments in contextual fear-memory and social motivation; this is the only study that evaluates a social component (163).

Finally, it is essential to consider that some deficits in tasks such as MWM could be attributed to other potential confounding factors, like motor dysfunction (164, 165).

#### 5.2.4 Interleukin 15

IL-15 belongs to the 4-α helix bundle family of cytokines, like IL-2. Although both bind to the IL-2/IL-15Rβγc receptor complex, it binds with high affinity to its specific IL-15α receptor (166). Through trans-signaling it is involved in the development, maintenance and activation of natural killer (NK) and long-lived CD8 memory cells, which promote apoptosis in cancer cells and cells infected with intracellular pathogens (167).

IL-15Rα is expressed in the adult CNS by nestin-positive cells in the SVZ and by mature neurons in the OB, while IL-15 is expressed by GFAP-positive cells in the SVZ and by immature neurons in the RMS (168, 169) (**Figure 2**, **Table 2**).

Gomez-Nicola et al. (168) demonstrated a negative effect of IL-15 KO on NSPC proliferation and maturation into DCX cells in the SVZ, with restoration after intraventricular injection of this cytokine. Regarding the cognitive aspects, it is vitally important to mention He et al. (170), who reported deficits in contextual fear conditioning in mice lacking IL-15Rα. Additionally, IL-15Rα^-/-^ mice show deficits in stone T-maze resolution and contextual fear memory tests. Unfortunately, no correlative studies of SVZ/RMS neurogenesis and OB-dependent behavior exist.

#### 5.2.5 Interferons alpha and beta

Interferons alpha and beta (IFN-α and IFN-β) are aptly named for their ability to truly interfere with viral replication. These interferons are produced mainly by macrophages and plasmacytoid dendritic cells. Virtually cells in the body express IFNAR1; upon cytokine-receptor ligation, the cell is prepared to fight a potential infection, which involves the induction of MHC-I expression, making the cell susceptible to CD8 cytotoxicity. Furthermore, several molecular mechanisms are activated to interfere with viral RNA or protein synthesis if the pathogen infects the cell (88).

IFN-α and IFN-β can be produced by neurons, microglia, and astrocytes (**Figure 2**). The former has been shown to modulate neuronal activity in specific brain regions, including the hypothalamus, thalamus, amygdala, and hippocampus (171, 172). *In vitro* studies with adult hippocampal neural stem cells and mature neurons have shown expression of both IFNR1 and IFNR2 (**Table 2**) (173).

Chronic i.v. administration of IFN-α has been shown to decrease cell proliferation within the DG, likely affecting NSPCs and reducing the survival of BrdU- and NeuN-labeled cells three weeks after the last administration (174). IL-1β may mediate these effects, as its levels increase in the hippocampus after injection of IFN-α. Notably, the inhibition of cell proliferation was reversed by i.c.v administration of IL-1ra (174).

Zheng et al. (173) conducted a comprehensive study encompassing *in vivo* and *in vitro* assessments of neurogenesis in both the hippocampus and SVZ as well as examining social behavior, reinforcing the findings above. In their investigation, a 4-week intraperitoneal treatment of C57BL6/J mice with IFN-α led to a reduction in cell proliferation and neuronal differentiation in the DG. In contrast, cell survival was not affected in this region. Similar effects were observed in the SVZ, where IFN-α administration decreased proliferation and reduced the population of MASH1 cells, indicating a negative impact on the self-renewal capacity of neural progenitor cells. Paradoxically, *in vitro* experiments demonstrated that only proliferation was affected, with no changes in differentiation observed. This decrease in proliferation was evident in cells obtained from both DG and SVZ. The study further established that the effect of IFN-α is direct on neural progenitors but could be amplified by the induction of synthesis of three inflammatory cytokines (IL-1β, IL-6, and TNF-α) by microglia (173, 175). The impact of IFN-β on neurogenesis remains unclear. However, Lum et al. (176) observed that culturing neurospheres isolated from SVZ with IFN-β inhibited proliferation and differentiation, similar to the effects of IFN-α. Additional studies on this cytokine are warranted.

The role of IFN-α and IFN-β in learning, memory, and social behavior is currently poorly understood. In a study by Hosseini et al. (177), a decrease in MWM performance was reported in both IFNAR^-/-^ and Ifnb^-/-^ mice. Another outcome was social behavior, as mice subjected to chronic IFN-α treatment showed poor performance in the Crawley social interaction test (173). These findings suggest type I interferons may impair behavior, but more studies are required for a comprehensive understanding.

#### 5.2.6 Granulocyte-macrophage colony-stimulating factor

There are three canonical members of myelopoiesis stimulating factors in mammals, i.e. granulocyte (G), monocyte/macrophage (M) and granulocyte/macrophage (GM) colony stimulating factors (CSF). All were shown to stimulate myeloid cell proliferation/differentiation *in vitro*. However, it is known that GM-CSF is not as important for steady-state myelopoiesis as previously thought, as it has rather restricted function for alveolar macrophages and some dendritic cells (178).

Instead, GM-CSF seems involved in promoting local and systemic inflammation. Its receptor (GM-CSFR) is made up of two chains, alpha and beta, the first holding the specificity for this cytokine. GM-CSF has now been involved in chronic inflammation, and various cell types, particularly activated T cells, produce it and act on resident macrophages, which release cytokines and reactive oxygen species (ROS), directly causing tissue damage. Activated granulocytes and tissue-resident DCs have also been proposed as targets of this molecule (178).

The GM-CSF GM-CSFRα is expressed in NSPCs, immature neurons, and mature neurons in the DG, but has only been reported on NSPCs in the SVZ (179, 180) (**Table 2**). *In vitro* treatment of adult neural stem cells with GM-CSF induces differentiation and maturation into neurons (179). *In vivo* studies have reported increased NSPCs proliferation in the DG and SVZ in a dose-dependent manner (181). However, Kiyota et al. (182) observed no changes in the number of DCX+ cells after chronic GM-CSF administration.

Both overexpression and knockdown of GM-CSFRα in the adult hippocampus resulted in slight improvement in MWM performance (180). Other studies suggest that the cytokine plays a more important role in memory than its receptor: GM-CSF knockout (GMko) mice exhibit impaired hippocampal-dependent memory, as demonstrated in MWM and contextual fear conditioning tests.

Similarly, Ahmed et al. (183) reported improved spatial memory in the radial-arm MWM after chronic GM-CSF administration. Although, Kiyota et al. (182) did not find any improvement in the same task with chronic GM-CSF treatment, this could be due to differences in dosage, as Ahmed et al. (183) administered 5 μg/kg, while Kiyota et al. (182) used 50 μg/kg body weight. These findings suggest a dose-dependent effect on spatial memory, such as the dose-response relationship observed in DG and SVZ neurogenesis (181). Furthermore, it cannot be ruled out that GM-CSF is acting through receptors for other cytokines, like IL-3 or IL-5 since it binds to their alpha chains (178).

#### 5.2.7 CCL-2, CCL-3 and CXCL1

CCL2 and CCL3, along with their respective receptors, CCR2 and CCR3/CCR5, are expressed by endothelial cells throughout the body and recruit diverse leukocyte populations (88).

Within the CNS, CCL2 is expressed in astrocytes and microglia (184; **Figure 2**). The expression of CCR2 and CCR3 at the mRNA and protein levels has been observed in different brain regions, such as the cerebral cortex, amygdala, thalamus, hypothalamus, and hippocampus (185–187). Furthermore, these receptors are expressed in the granule and hilar cells of the DG and in the granule cells of the OB (187). Ji et al. (188) also detected the expression of CCR2 and CCR5 in NSPCs from adult SVZ rats cultured in neurospheres (**Table 2**).

Regarding neurogenesis, Liu et al. (189) observed that CCL2 increased the differentiation and migration of SVZ-derived NSPCs *in vitro* (**Table 3**). In contrast, Gordon et al. (186) found that CCL2 inhibited the differentiation of adult rat SVZ progenitors into MAP-2 (early neurons) or RIP (oligodendrocytes) *in vitro* while strongly stimulating GFAP cell production. The authors maintain that they allowed the cells to differentiate seven days longer than Liu et al. (189). Additionally, differences between species and variation in doses used (lower in Liu et al., higher in Gordon et al.) must be considered when interpreting these contrasting results.

Notably, CXCL1 favored differentiation towards all three neural types, with a notable preference for neurons dominating the population at the stimulation end (186).

Regarding learning and memory, Marciniak et al. (190) reported that i.c.v. chronic administration of CCL3 significantly impairs long-term memory in passive avoidance, accompanied by a reduction in LTP (long-term potentiation). Unfortunately, changes in neurogenesis within the DG were not evaluated in this work.

#### 5.2.8 Eotaxin (CCL11)

Eosinophils, basophils and Th2 cells are recruited to extracerebral tissues by eotaxin, which acts through CCR3. Endothelial cells that express CCR3 are also the main producers of the chemokine (88).

The only study related to this chemokine and neurogenesis is that of Villeda et al. (191), who conducted an elegant study with a cohort of mice. They showed that systemic administration of humoral (non-cellular) components of the blood of old animals decreased differentiation of young animals in the DG, as assessed by the generation of DCX cells. They showed that CCL11 was one of the main molecules affecting both neurogenesis and LTP within the DG, which were reversed by local administration of specific monoclonal antibodies. Interestingly, they found impairment in contextual fear conditioning in the radial arm water maze by i.p. CCL11 administration.

#### 5.2.9 CXCL12

The chemokine CXCL12 (SDF-1) and its receptor, CXCR4, participate in the trafficking of B lymphocytes and plasma cells to lymph nodes and bone marrow, respectively (88). Chemokine mRNA has been identified in neurons throughout the brain, including the cerebral cortex and hippocampus. Additionally, the mRNA coding for CXCR4 has been reported in the hippocampus, ependymal layers of the ventricles, and the OB (192). The chemokine and its receptors, CXCR4 and CXCR7, are expressed on the endothelial membrane and in several types of brain cells, including astrocytes, microglia, and neurons. It should be noted that the receptors are coexpressed in the same cell (193).

Abe et al. (194) reported that CXCL12/CXCR7 regulates the maturation and survival of new granule neurons in the DG of mice. To demonstrate this, they removed CXCL12 and CXCR7 from the granule cell layer, resulting in fewer proliferating cells (BrdU). Although the number of BrdU/NeuN was not affected, there was disorganization of the DG cell layers and dendritic growth of immature neurons.

Trousse et al. (195) observed in CXCL12^+/-^ mice a reduced number of Ki67 cells without changes in DCX cell counts. In CXCR7^+/-^ mice, they observed the opposite: no changes in Ki67 and decreased DCX cells. Furthermore, they noted that CXCL12^+/-^ mice presented an impairment in spatial memory, which was evaluated in the MWM. These findings emphasize the role of CXCL12 or its receptor not only in neurogenesis but also in learning and memory.

### 5.3 Adaptive response effector/inflammatory cytokines

#### 5.3.1 Interferon gamma

The discovery of interferon-γ (IFN-γ) dates to 1965 when it was identified as a protein produced by leukocytes in response to phytohemagglutinin, but its name was not formally coined until 1980 (196, 197). As Billiau and Matthys highlighted in 2009 (198), this cytokine was named for its role as an antiviral factor, but has broader regulatory functions on leukocytes and other non-immune cells. In fact, IFN-γ and its receptor are not structurally or functionally related to type I interferons.

This cytokine is essential in antigen processing and presentation because it activates genes related to these processes in accessory cells. Furthermore, it has antitumor effects by inducing cell cycle arrest and apoptosis, activates the antimicrobial effector functions of phagocytes and NK lymphocytes, shifts T helper cells toward a Th1 phenotype, and strongly promotes inflammation by enhancing leukocyte trafficking, myeloid cell production of IL-12 and chemokines, as well as the expression of adhesins by endothelial cells. It is produced by innate (NK) and adaptive lymphocytes (CD3+CD4+ and CD3+CD8+) after induction by IL-12 or IFN-γ itself (88).

Although generally undetectable in the CNS, IFN-γ is expressed in response to infections and other disorders (199). Despite the lack of information on the expression of its receptors in the DG, there are reports on its action. For example, Barón et al. (200) used a transgenic mouse model that expressed low constitutive levels of IFN-γ in the adult brain. They observed an increase in early-stage cell proliferation and differentiation in the hippocampus of three-month-old mice. Notably, these brains had elevated levels of IL-6, TNF-α, and BDNF mRNA; therefore, its action could be indirect. Similarly, Campos et al. (201) demonstrated that IFN-γ KO mice have reduced numbers of DCX cells in the DG. A conflicting result came from another study with IFN-γ KO mice, which showed increased differentiation of early progenitor cells in the same region (202). Likewise, a single i.c.v. Injection of IFN-γ reduced the number of BrdU and DCX/NeuN cells in this region due to increased apoptosis of immature cells, microglia-derived inflammatory cytokines, and nitric oxide, a phenotype hindered by the antibiotic minocycline or with Ruxolitinib, an inhibitor of JAK/STAT1 signaling (203).

In the SVZ, expression of both receptors, IFNR1 and IFNR2, has been demonstrated in NSPCs (204; **Table 2**). Three *in vitro* studies with mouse cells showed increased differentiation of NSPCs from the SVZ, although conflicting results were reported regarding proliferation (133, 176, 205). Li et al. (206) observed that neurospheres from IFN-γ^−/−^ mice exhibited increased proliferation and differentiation into neurons and oligodendrocytes and reduced differentiation into astrocytes. When IFN-γ was added to the culture medium, these effects were reversed. The same study demonstrated that IFN-γ KO is associated with increased proliferation *in vivo*. Pereira et al. (207) suggested a similar effect, and reported that i.c.v. administration of IFN-γ reduces the proliferation and differentiation of SVZ cells and decreases the survival of new neurons in the OB of rats.

Experimental data and controversy hardly support the role of IFN-γ in memory and learning. Genetically modified mice with limited brain expression of IFN-γ, which does not cause cellular damage or infiltration, demonstrated better MWM performance than wild-type animals (200). However, Monteiro et al. (202) found that IFN-γ KO mice performed better on MWM and novel object recognition tests. In social behavior there is only one work by Filiano et al. (208), who reported that mice lacking IFN-γ expression showed reduced social interaction.

These results suggest that basal levels of the cytokine are necessary for neurogenesis in the SGZ and SVZ and memory because high levels or absence of this cytokine impair them.

#### 5.3.2 Interleukins 4 and 13

Interleukins 4 and 13 are produced by Th2 CD4+ cells. They share the IL-4Rα chain and participate in several effector functions. IL-4 has broad actions on different cells and can be produced by multiple cell types in addition to Th2 cells, including CD4+NK1.1+ natural T cells (NKT), macrophages, eosinophils, basophils, mast cells, and type 2 innate lymphoid cells (ILC2). It is a fundamental cytokine for antibody production, but is also involved in Th2 inflammation, fibrosis, allergic reactions, and antitumor activity (209).

IL-4Rα is highly expressed in the cortex and hippocampus in GABAergic and glutamatergic neurons (**Table 2**). Its deficiency has been linked to the reduction of synaptic vesicles in the hippocampus, indicating that this receptor plays a crucial role in synaptic activity within this region (210). Regarding neurogenesis, we found no studies on the expression of IL-4Rα in young adult mice. However, there is a report investigating this receptor in NSPCs of 12-month-old wild-type C3B6F1 mice, but they did not find it (211).

Higher levels of IL-4 have been reported in the peripheral and CNS as a result of genetic restriction of the T cell repertoire to a foreign antigen (OVA) in Balb/c mice; this increase was negatively correlated with the number of Ki-67 and DCX cells, but no direct effect of the cytokine was shown *in vivo*. In this study, the researchers further analyzed the impact of cytokine on hippocampal NSPC cultures. They observed reduced proliferation, a response that appears to be dose- and time-dependent (94). In contrast, Zhang et al. (212) observed no changes in proliferation, differentiation, or survival in NSPCs of the DG due to IL-4 or IL-4Rα KO. However, they used CX3CR1 KO mice and as mentioned above, this receptor has a vital role in neurogenesis. *In vitro*, Guan et al. (213) did not find an increase in migration in neurospheres obtained from SVZ.

IL-4 and IL-13 have been linked to memory and learning, as evidenced by an increase in their levels within the meninges and hippocampus when a mouse performs a spatial task. Furthermore, mice lacking IL-4, IL-13, or IL-4Rα exhibit deficits in MWM resolution and contextual fear conditioning (97, 210, 214–216). Moon et al. (217) reported no changes in IL-4 KO mice in either MWM or novel object recognition tests, and state that differences due to active/inactive cycling between their study and that of Derecki et al. (216) could explain the differences. Finally, this study was the only one that explored the effect of lack of IL-4; in this case an increase in social exploration was observed, suggesting an inhibitory function of the cytokine (217).

#### 5.3.3 Interleukins 17 and 23

The IL-17 cytokine family includes six members: IL-17A, B, C, D, E and F, produced by Th17 (inflammatory) lymphocytes and Tγ/δ and CD8 cells (88). Isoform A is the most active and indirectly mediates intense neutrophil-mediated inflammation by enhancing the production of IL-1β, IL-6 and G-CSF by tissue-resident cells. It induces a much more powerful inflammatory response than an initial innate aggression, thus closing a positive feedback loop to attack bacteria and fungi.

In the DG, IL-17 mRNA is mainly expressed by astrocytes and, to a lesser extent, by microglia and NSPCs (218; **Figure 2**). IL-17 KO mice showed increased differentiation, maturation, and survival of neurogenic cells in the DG (218). Surprisingly, this was accompanied by a decrease in several cytokines such as IFN-γ, TNF-α, IL-1β, and IL-12, suggesting that these effects could be indirect (218). Consistent with these findings, IL-17A administration decreased the relative gene expression of PCNA, indicating a reduction in cell proliferation in this area (219). In apparent contradiction, i.p. administration of anti-IL-17A antibodies reduced the number of DCX cells (220). However, it is unclear whether IL-17 crosses the BBB to access the neurogenic niche directly or whether its effect is indirect. Therefore, further studies are necessary to identify the mechanism of the effect of IL-17 on neurogenesis. Regarding memory and learning, IL-17 KO mice showed no changes in memory performance, as assessed in MWM and Y-maze tests (221).

Interleukin-23 was discovered almost 20 years ago; is a member of the IL-12 family, formed by the p19 and p40 subunits, and is a critical promoter of Th17 differentiation and proliferation through interaction with IL-12Rβ1 and the specific IL-23 receptor (222). The study by Willinger and Turgman (220) is the only exploration of the role of IL-23 in social behavior and memory. They reported that i.p. administration of anti-IL-23 reduced social interaction with conspecifics. Furthermore, a decrease in the number of DCX cells was observed in the DG (220). The specific effect of IL-23 on neurogenesis in SVZ-BO is still unknown, but due to its role on Th17 cells it could be acting through them.

It is essential to highlight the need for more research on the cytokines IL-17 and IL-23 in neurogenesis and behavior, mainly due to the abundance of Tγ/δ in the meningeal lymphatic network and the SLYM (103, 104).

### 5.4 Regulatory cytokines

#### 5.4.1 Interleukin 2

Interleukin-2 (IL-2) was discovered in 1976 by Morgan, Ruscetti and Gallo. They demonstrated that a “conditioned medium” obtained from normal human lymphocytes stimulated with phytohemagglutinin induced selective proliferation and maintenance of T cells, revealing an autocrine stimulator. It is understood that both IL-2 and the alpha chain of its receptor (IL-2Rα or CD25) are produced exclusively by T cells immediately after antigenic stimulation in secondary lymphoid tissues. Regulatory T cells (Treg) represent an exception, constitutively expressing the full form of the receptor (IL-2Rα/β/γc) and deficient levels of cytokine. This unique expression pattern strongly associates IL-2 with the regulation of the immune response (88).

The cytokine and its receptor have been identified in several regions of the CNS, including the prefrontal cortex, striatum and hippocampus (223, 224). Both IL-2Rα and IL-2β have been found in mature neurons of the DG and SVZ (**Table 2**).

Due to the limited number of studies, the role of IL-2 in neurogenesis has not yet been elucidated. Beck et al. (225) reported that mice lacking IL-2 exhibited increased differentiation of neurons (BrdU/βIIITubulin cells) in the DG, suggesting a modulatory effect, since IL-2 deletion also resulted in elevated levels of IL-12, IL-15, IP-10 (CxCL10), and MCP-1 (CCL2) in the hippocampus.

Regarding memory and learning, there are very few results that allow conclusions to be drawn. On the one hand, Petitto et al. (226) reported that mice lacking IL-2 showed impaired resolution of MWM tasks. Still, a later study by the same group found that deletion of the standard IL-2/IL-15β chain from the receptor did not influence performance in the MWM, leaving doubt about the role of IL-2 (227). These findings suggest that IL-2/IL-2Rα signaling is necessary for normal spatial memory. Finally, Wu et al. (228) found that mice with a knockout IL-2Rγc gene (involved in IL-2 signaling) had improved contextual fear conditioning. However, the receptor gamma chain is shared with other cytokine receptors, i.e. IL-4, IL-7, IL-9, IL-15, and IL-21 (88); Therefore, it is not clear which cytokine is involved in the phenomenon.

#### 5.4.2 Interleukin 10

Interleukin-10 is a potent immune mediating cytokine with versatile functions, initially described as a product of activated CD4 Th2 cells and termed cytokine synthesis inhibitory factor (229). It is now known that a large subset of innate and adaptive immune cells, including dendritic cells, macrophages, eosinophils, NK cells, T cells, and B cells, can produce IL-10 (230). IL-10 is generally considered an immunosuppressive cytokine, often referred to as the “brake” of the immune system; however, IL-10 can also act as an activator of B cells and CD8 T cells (231).

In the CNS, IL-10 is produced by microglia, astrocytes, and neurons (**Figure 2**). It exerts various biological effects in the brain, such as limiting the synthesis of pro-inflammatory molecules, mediating neuroprotection, and modulating synaptic structure and activity (232, 233).

In hippocampal neurogenesis, a recent study by Sánchez-Molina et al. (234) with transgenic mice that overexpress IL-10 did not find significant differences in proliferation. However, they reported a notable decrease in the survival and differentiation of newborn NSPCs. Notably, IL-10R is not expressed in these cells, suggesting an indirect effect of the cytokine on this process. Sánchez-Molina et al. (234) discuss that this indirect effect could be mediated by CX3CR1, since overexpression of IL-10 leads to a reduction of the receptor, which decreases neurogenesis (75–80).

IL-10R1 expression in the SVZ is limited to Nestin cells in the dorsal subregion, with no expression observed in the ventral subregion (235; **Table 2**). After an acute i.c.v. After IL-10 administration, the overall population of proliferating cells (BrdU) remained stable. However, a specific decrease in BrdU/Nestin cells indicated a decrease in NSPC proliferation.

Furthermore, Nestin/Mash1 cells increased, but simultaneously, differentiation (DCX and PSA-NCAM cells) decreased (235).

As mentioned above, direct injection of Treg cells (previously stimulated with IL-2 and anti-CD28) into the SVZ enhanced NSPC proliferation; this effect was mediated by IL-10 (95). Therefore, because there is an increase in Nestin/Mash1 but a decrease in proliferation and differentiation, these results suggest that IL-10 may play a role in arresting lineage progression and potentially contribute to maintain theNSPC reservoir, as indicated by Pérez Ascencio et al. (235).

The described observations align with the assessment of NSPC survival in the OB, where acute administration of IL-10 and, paradoxically, its absence (in IL-10 knockout mice) reduce BrdU/DCX and BrdU/NeuN cell populations. These findings suggest that IL-10 could inhibit NSPC proliferation in the dorsal SVZ and their maturation in the OB (235). Guan et al. (213) reported a slight increase in migration of neurospheres obtained from the SVZ *in vitro*. It should be noted that there is a strict regionalization of the neurogenic niches in the ventral and dorsal subregions of the SVZ in terms of gene expression, with the dorsal area being the gateway to the RMS and the final objective, the OB (236).

In an interesting study by Sánchez-Molina et al. (234) using a model of chronic IL-10 overproduction, a deficit in spatial memory was demonstrated. Paradoxically, transgenic mice with reduced IL-10 expression in the brain also showed impairments in MWM performance (237). These contradictory findings underscore the intricate role of IL-10 in modulating cognitive functions.

#### 5.4.3 Tumor growth factor beta

Like IL-10, TGF-β family members are considered master immunosuppressive regulators. TGF-β1 inhibits the proliferation of T and B cells and the activation of macrophages, and also has other functions, such as inducing class switching in B cells to produce IgA, promoting the differentiation of Th0 lymphocytes into Treg or Th17 (along with IL-6), and facilitating the production of extracellular matrix, specifically collagen by fibroblasts. Consequently, TGF-β plays a crucial role in tissue repair, scar formation, fibrosis, apoptosis, cell motility, and angiogenesis. It is primarily produced by regulatory T cells and macrophages, although other cell types can also secrete it (882; 238, 239).

TGF-β receptors are expressed in multiple brain regions, including the cortex, midbrain, cerebellum, brainstem, and hippocampus. Microglia are the main source of TGF-β1 in the CNS, while the producers of TGF-β2 and β3 in murine brains are yet unknown (240, 241). Wachs et al. (242) demonstrated the expression of Nestin neural stem cells from DG and SVZ that express mRNA for all three receptors, TGF-βR1, TGF-βR2, and TGF-βR3 (**Table 2**).

When TGF-β1, but not TGF-β2, was added to cultures containing these NSPCs, a dose-dependent reduction in their proliferation, arresting the cells in G0/G1 was observed (242; **Table 1**). Buckwalter et al. (243) and Kandasamy et al. (244) reported that transgenic mice overexpressing TGF-β1 reduced the proliferation, differentiation, and maturation of NSPC cells in the DG. However, in the second work an increase in NSPC survival was observed. Furthermore, chronic infusion of TGF-β1 for seven days into the brain ventricles inhibits NSPC proliferation in both the hippocampus and SVZ (242). This inhibitory effect persists four weeks after the last administration, with no changes observed in cell differentiation and maturation. However, Mathieu et al. (245) reported conflicting results, since rats injected with adenoviral vectors expressing TGF-β in the SVZ exhibited increased differentiation (BrdU/DCX cells) three weeks later. Discrepancies may arise due to differences in the vector used for transgenesis or in the mammalian species studied. Interestingly, deletion of TGF-βR2 does not alter neuroblast exhaustion, leaving open the question of how TGF-β affects neurogenesis (246).

Shih et al. (247) reported that inhibition of TGF-β1 signaling through PHFR1 KO affected MWM performance. Similarly, Bedolla et al. (240) found that deletion of the microglial-*Tgfb1* gene affects performance in the Barnes maze. Interestingly, overexpression of the cytokine during adulthood increases social interaction in mice, underscoring the context-dependent effects of TGF-β on brain function (248).

## 6 Discussion

Although the initial inspiration to analyze the role of cells and cytokines in behavior, learning and memory was their alterations due to infectious and non-infectious diseases, we soon realized that their role in non-pathological conditions was unclear. As mentioned at the beginning, the extraordinary reviews cover part of the topics discussed here. Still, many include different species (humans, rodents and others) at different ages and often in pathological conditions. However, these works gave us great ideas to define the limits of this review to reduce heterogeneity and to seek conclusions about the role of the immune system within the CNS, especially in two fundamental areas for neuroplasticity through neurogenesis: the DG and the SVZ. We found some data that can lead to conclusions.

### 6.1 Virtually all resident but few “invader” cells participate in neurogenesis under non-pathological conditions

All resident and a few extracerebral cell types influence neurogenesis and behavior primarily through the production of neurotrophic factors and cytokines. Two extracerebral immune cells are essential for neurogenesis in a non-diseased brain. The first is Ly6Chi monocytes, which have positive effects, possibly through cytokine production. Resident neuronal and glial cells can attract them by secreting CCL2. Furthermore, CD4+ T cells participate in neurogenesis and behavior, providing neurotrophic factors, such as BDNF or cytokines. Interestingly, their antigenic specificity influences their function, and those that are autoreactive could have a positive function.

The crucial resident immune cells are microglia, which are pro-neurogenic if quiescent, probably through scavenger-like activity, and provide neurotrophic factors and adequate levels of some cytokines. However, if activated, they produce high levels of pro-inflammatory cytokines, which may inhibit neurogenesis, with potential effects on learning, memory and social behavior.

### 6.2 Are cytokines direct causative factors of neurogenesis and behavior?

**Figure 3:**
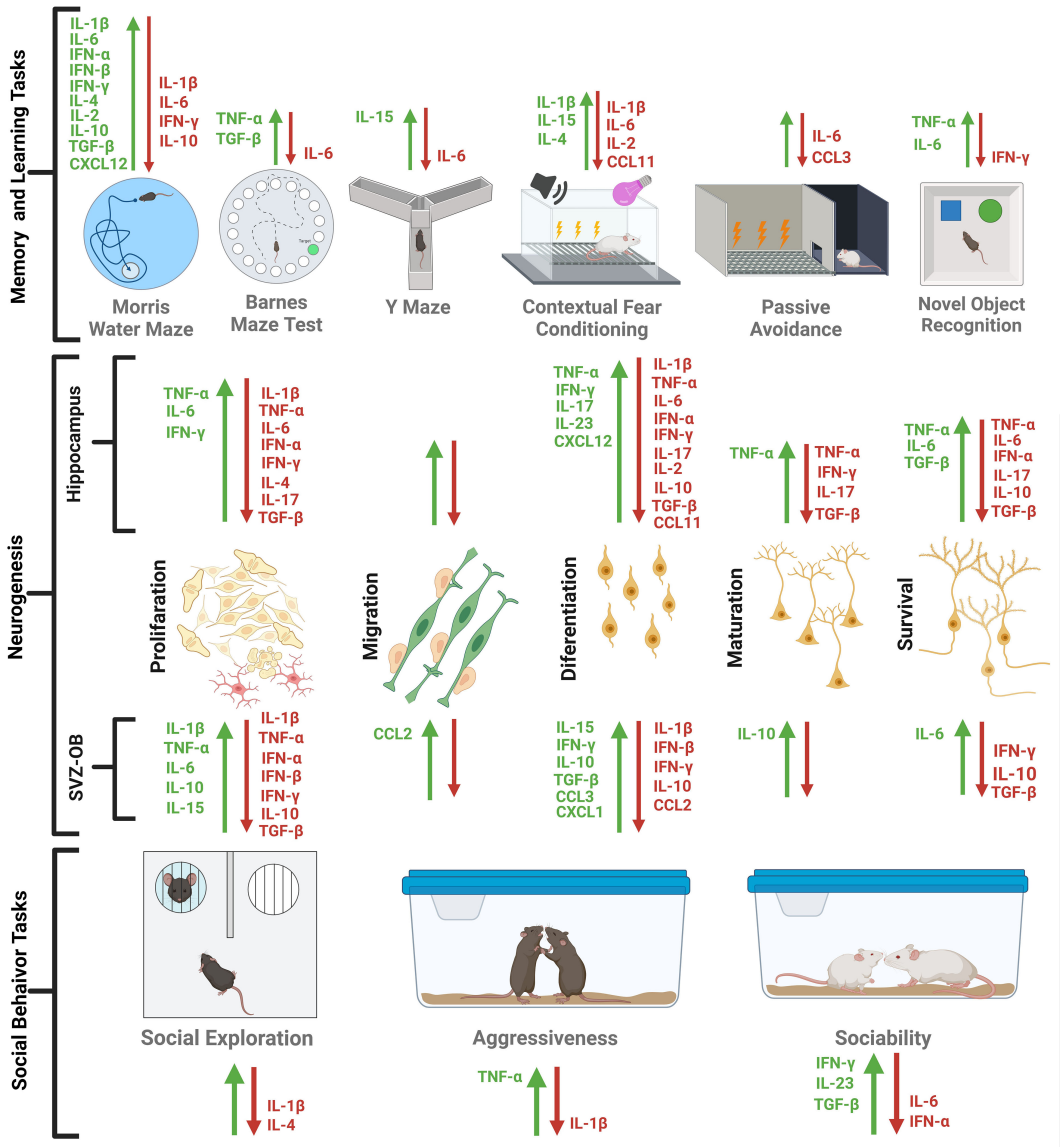
Effect of cytokines and chemokines on hippocampal and SVZ neurogenesis and on memory/learning or social behavior. Green arrows indicate better performance in behavioral tests or increase in proliferation, migration, differentiation, maturation or survival of the new neurons generated, while the red arrows indicate a worsening of the same phenomena. The scheme was made in BioRender License *VX26YDSAAS.*.

We conducted this review to identify key actions of cytokines on neurogenesis and their cognitive implications. We constructed **Table 1** and **Figure 3** to show the main findings, and they do so to some extent. Extensive research has suggested that some inflammatory cytokines, such as IL-1β, IL-6, TNF-α, IFN-α, and IFN-γ, but also the anti-inflammatory IL-10 and TGF-β, inhibit neurogenesis. However, these molecules can have both stimulatory and inhibitory effects (**Table 1**). Part of the reason is that there is a dose-dependent effect on cell survival, differentiation, or proliferation, as shown for TNF-α.

Remarkably, much of the data comes from KO models, with few exceptions of tissue-specific, cell type-specific, or time-specific transgene expression; therefore, they are inconclusive, as the animals lacked or overexpressed the cytokine throughout their bodies and lives.

Several studies reviewed here strongly suggest that cytokines interact and are co- produced by single-cell types. Therefore, the effect of a cytokine may well reflect the simultaneous or subsequent actions of a series of molecules.

Although there is strong support for the importance of chemokines in neurogenesis due to the presence of their receptors in progenitor, immature, and mature neurons in both the DG and SVZ (see **Table 2**), direct evidence for their role is scarce. However, they have been involved in cell migration, differentiation and maturation (**Table 3**). There is no evidence for chemokine production by CNS resident cells other than CCL2 and Cx3CL1 (**Figure 2**).

### 6.3 Cytokines or their receptors?

In some cases, receptors, not cytokines, were shown to be the key factors in neurogenesis. Two examples are fractalkine and TNF-α. In the first case, the lack of the receptor makes the microglia amoeboid very active and more inflammatory, while the absence of the cytokine had no effect. For TNF-α, it was elegantly shown that the two receptors act in different and almost opposite ways: TNFR2 induces the proliferation of NSPCs and TNFR1 causes them to return to an inactive state, thus maintaining the pool of potential precursors. In **Table 2**, most NSPCs and mature neurons in the DG have been directly studied for receptor expression, while much remains to be done in the SVZ. This is relevant because, as explained at the beginning, the proliferation of progenitors is not always related to neurogenesis; in fact, it may indicate a deviation towards the genesis of astrocytes or oligodendrocytes.

### 6.4 Behavioral changes caused by immune cells and cytokines could be due to factors non-related to neurogenesis

The expression of cytokine and chemokine receptors in mature neurons in the hippocampus and olfactory bulb (**Tables 1**, **2**) raises the possibility that immune molecules directly regulate behavior. In this review, we highlight some studies in which cytokines (e.g., IL-1β, CCL-3) were injected directly into the brain parenchyma, inducing memory impairment (114, 190). Particularly, in the few studies in which neurogenesis and behavior were evaluated simultaneously, concordant results emerged. For example, Sánchez-Molina et al. (234) demonstrated that transgenic overexpression of IL-10 in the hippocampus enhanced DG neurogenesis and improved spatial memory performance, directly linking cytokine levels, neurogenesis, and behavior. These studies underscore that, under strictly controlled experimental conditions, immune-mediated neurogenesis can be correlated with behavioral outcomes.

While these findings suggest a link, establishing a causal mechanism requires further research in this field, because the effects on behavior could arise, for example, through cytokine-driven alterations in synaptic plasticity (18) or neuroendocrine signaling (249) rather than directly on neurogenesis.

## References

[1] Dantzer R Konsman JP Bluthé RM Kelley KW. Neural and humoral pathways of communication from the immune system to the brain: parallel or convergent? Auton Neurosci. (2000) 85:60–5. doi: 10.1016/S1566-0702(00)00220-4 11189027

[2] Dantzer R. Neuroimmune interactions: from the brain to the immune system and vice versa. Physiol Rev. (2018) 98:477–504. doi: 10.1152/physrev.00039.2016 29351513 PMC5866360

[3] Ziemssen T Kern S. Psychoneuroimmunology–cross-talk between the immune and nervous systems. J Neurol. (2007) 254 Suppl 2:II8–11. doi: 10.1007/s00415-007-2003-8 17503136

[4] Schwartz M. Neuroimmunity. In: A new science that will revolutionize how we keep our brains healthy and young. Yale University Press, New Haven and London (2015).

[5] Al-Onaizi M Al-Khalifah A Qasem D ElAli A. Role of microglia in modulating adult neurogenesis in health and neurodegeneration. Int J Mol Sci. (2020) 21:6875. doi: 10.3390/ijms21186875 32961703 PMC7555074

[6] Asamu MO Oladipo OO Abayomi OA Adebayo AA. Alzheimer’s disease: The role of T lymphocytes in neuroinflammation and neurodegeneration. Brain Res. (2023) 1821:148589. doi: 10.1016/j.brainres.2023.148589 37734576

[7] Altman J Das GD. Autoradiographic and histological evidence of postnatal hippocampal neurogenesis in rats. J Comp Neurol. (1965) 124:319–35. doi: 10.1002/cne.901240303 5861717

[8] Doetsch F García-Verdugo JM Alvarez-Buylla A. Cellular composition and three-dimensional organization of the subventricular germinal zone in the adult mammalian brain. J Neurosci. (1997) 17:5046–61. doi: 10.1523/jneurosci.17-13-05046.1997 PMC6573289 9185542

[9] Domínguez-Rivas E Ávila-Muñoz E Schwarzacher SW Zepeda A. Adult hippocampal neurogenesis in the context of lipopolysaccharide-induced neuroinflammation: A molecular, cellular and behavioral review. Brain Behav Immun. (2021) 97:286–302. doi: 10.1016/j.bbi.2021.06.014 34174334

[10] Haroon E Raison CL Miller AH. Psychoneuroimmunology meets neuropsychopharmacology: translational implications of the impact of inflammation on behavior. Neuropsychopharmacology. (2012) 37:137–62. doi: 10.1038/npp.2011.205 PMC3238082 21918508

[11] Lledo PM Valley M. Adult olfactory bulb neurogenesis. Cold Spring Harb Perspect Biol. (2016) 8:a018945. doi: 10.1101/cshperspect.a018945 27235474 PMC4968158

[12] Salvador AF de Lima KA Kipnis J. Neuromodulation by the immune system: a focus on cytokines. Nat Rev Immunol. (2021) 21:526–41. doi: 10.1038/s41577-021-00508-z 33649606

[13] Toda T Parylak SL Linker SB Gage FH. The role of adult hippocampal neurogenesis in brain health and disease. Mol Psychiatry. (2019) 24(1):67–87. doi: 10.1038/s41380-018-0036-2 29679070 PMC6195869

[14] Borsini A Zunszain PA Thuret S Pariante CM. The role of inflammatory cytokines as key modulators of neurogenesis. Trends Neurosci. (2015) 38:145–57. doi: 10.1016/j.tins.2014.12.006 25579391

[15] Chintamen S Imessadouene F Kernie SG. Immune regulation of adult neurogenic niches in health and disease. Front Cell Neurosci. (2021) 14:571071. doi: 10.3389/fncel.2020.571071 33551746 PMC7855589

[16] Das S Basu A. Inflammation: a new candidate in modulating adult neurogenesis. J Neurosci Res. (2008) 86:1199–208. doi: 10.1002/jnr.21585 18058947

[17] Kohman RA Rhodes JS. Neurogenesis, inflammation and behavior. Brain Behav Immun. (2013) 27:22–32. doi: 10.1016/j.bbi.2012.09.003 22985767 PMC3518576

[18] Yirmiya R Goshen I. Immune modulation of learning, memory, neural plasticity and neurogenesis. Brain Behav Immun. (2011) 25:181–213. doi: 10.1016/j.bbi.2010.10.015 20970492

[19] Camina E Güell F. The neuroanatomical, neurophysiological and psychological basis of memory: current models and their origins. Front Pharmacol. (2017) 8:438. doi: 10.3389/fphar.2017.00438 28713278 PMC5491610

[20] Kim WB Cho JH. Encoding of contextual fear memory in hippocampal-amygdala circuit. Nat Commun. (2020) 11:1382. doi: 10.1038/s41467-020-15121-2 32170133 PMC7069961

[21] Sharma S Rakoczy S Brown-Borg H. Assessment of spatial memory in mice. Life Sci. (2010) 87:521–36. doi: 10.1016/j.lfs.2010.09.004 PMC6457258 20837032

[22] Lisman JE. Relating hippocampal circuitry to function: recall of memory sequences by reciprocal dentate-CA3 interactions. Neuron. (1999) 22:233–42. doi: 10.1016/s0896-6273(00)81085-5 10069330

[23] Basu J Siegelbaum SA. The Cortico Hippocampal circuit, synaptic plasticity, and memory. Cold Spring Harb Perspect Biol. (2015) 7:a021733. doi: 10.1101/cshperspect.a021733 26525152 PMC4632668

[24] Eichenbaum H. The hippocampus and declarative memory: cognitive mechanisms and neural codes. Behav Brain Res. (2001) 127:199–207. doi: 10.1016/s0166-4328(01)00365-5 11718892

[25] Baum MJ Kelliher KR. Complementary roles of the main and accessory olfactory systems in mammalian mate recognition. Annu Rev Physiol. (2009) 71:141–60. doi: 10.1146/annurev.physiol.010908.163137 18817511

[26] Kaidanovich-Beilin O Lipina T Vukobradovic I Roder J Woodgett JR. Assessment of social interaction behaviors. J Vis Exp. (2011) 25:2473. doi: 10.3791/2473 PMC3197404 21403628

[27] Ko J. Neuroanatomical substrates of rodent social behavior: the medial prefrontal cortex and its projection patterns. Front Neural Circuits. (2017) 11:41. doi: 10.3389/fncir.2017.00041 28659766 PMC5468389

[28] Kim Y Venkataraju KU Pradhan K Mende C Taranda J Arganda-Carreras I. Mapping social behavior-induced brain activation at cellular resolution in the mouse. Cell Rep. (2015) 10:292–305. doi: 10.1016/j.celrep.2014.12.014 25558063 PMC4294964

[29] Firestein S. How the olfactory system makes sense of scents. Nature. (2001) 413:211–8. doi: 10.1038/35093026 11557990

[30] Anacker C Hen R. Adult hippocampal neurogenesis and cognitive flexibility - linking memory and mood. Nat Rev Neurosci. (2017) 18:335–46. doi: 10.1038/nrn.2017.45 PMC6261347 28469276

[31] Denoth-Lippuner A Jessberger S. Formation and integration of new neurons in the adult hippocampus. Nat Rev Neurosci. (2021) 22:223–36. doi: 10.1038/s41583-021-00433-z 33633402

[32] Lazarov O Hollands C. Hippocampal neurogenesis: Learning to remember. Prog Neurobiol. (2016) 140:1–18. doi: 10.1016/j.pneurobio.2015.12.006 PMC4828289 26855369

[33] Lieberwirth C Pan Y Liu Y Zhang Z Wang Z. Hippocampal adult neurogenesis: Its regulation and potential role in spatial learning and memory. Brain Res. (2016) 1644:127–40. doi: 10.1016/j.brainres.2016.05.015 PMC5064285 27174001

[34] Berg DA Bond AM Ming GL Song H. Radial glial cells in the adult dentate gyrus: what are they and where do they come from? F1000Res. (2018) 7:277. doi: 10.12688/f1000research.12684.1 29568500 PMC5840617

[35] Epp JR Silva Mera R Köhler S Josselyn SA Frankland PW. Neurogenesis-mediated forgetting minimizes proactive interference. Nat Commun. (2016) 7:10838. doi: 10.1038/ncomms10838 26917323 PMC4773435

[36] Kempermann G Brandon EP Gage FH. Environmental stimulation of 129/SvJ mice causes increased cell proliferation and neurogenesis in the adult dentate gyrus. Curr Biol. (1998) 8:939–42. doi: 10.1016/s0960-9822(07)00377-6 9707406

[37] Lee J Seroogy KB Mattson MP. Dietary restriction enhances neurotrophin expression and neurogenesis in the hippocampus of adult mice. J Neurochem. (2002) 80:539–47. doi: 10.1046/j.0022-3042.2001.00747.x 11905999

[38] Mak GK Enwere EK Gregg C Pakarainen T Poutanen M Huhtaniemi I. Male pheromone-stimulated neurogenesis in the adult female brain: possible role in mating behavior. Nat Neurosci. (2007) 10(8):1003–11. doi: 10.1038/nn1928 17603480

[39] Tanapat P Hastings NB Reeves AJ Gould E. Estrogen stimulates a transient increase in the number of new neurons in the dentate gyrus of the adult female rat. J Neurosci. (1999) 19:5792–801. doi: 10.1523/jneurosci.19-14-05792.1999 PMC6783062 10407020

[40] van Praag H Shubert T Zhao C Gage FH. Exercise enhances learning and hippocampal neurogenesis in aged mice. J Neurosci. (2005) 25:8680–5. doi: 10.1523/jneurosci.1731-05.2005 PMC1360197 16177036

[41] Birch AM McGarry NB Kelly AM. Short-term environmental enrichment, in the absence of exercise, improves memory, and increases NGF concentration, early neuronal survival, and synaptogenesis in the dentate gyrus in a time-dependent manner. Hippocampus. (2013) 23:437–50. doi: 10.1002/hipo.22103 23460346

[42] Zhang XQ Mu JW Wang HB Jolkkonen J Liu TT Xiao T. Increased protein expression levels of pCREB, BDNF and SDF-1/CXCR4 in the hippocampus may be associated with enhanced neurogenesis induced by environmental enrichment. Mol Med Rep. (2016) 14:2231–7. doi: 10.3892/mmr.2016.5470 27432087

[43] Clelland CD Choi M Romberg C Clemenson GD Jr Fragniere A Tyers P. A functional role for adult hippocampal neurogenesis in spatial pattern separation. Science. (2009) 325:210–3. doi: 10.1126/science.1173215 PMC2997634 19590004

[44] Diederich K Bastl A Wersching H Teuber A Strecker JK Schmidt A. Effects of different exercise strategies and intensities on memory performance and neurogenesis. Front Behav Neurosci. (2017) 11:47. doi: 10.3389/fnbeh.2017.00047 28360847 PMC5352691

[45] Sahay A Scobie KN Hill AS O’Carroll CM Kheirbek MA Burghardt NS. Increasing adult hippocampal neurogenesis is sufficient to improve pattern separation. Nature. (2011) 472:466–70. doi: 10.1038/nature09817 PMC3084370 21460835

[46] Dupret D Revest JM Koehl M Ichas F De Giorgi F Costet P. Spatial relational memory requires hippocampal adult neurogenesis. PloS One. (2008) 3:e1959. doi: 10.1371/journal.pone.0001959 18509506 PMC2396793

[47] Saxe MD Battaglia F Wang JW Malleret G David DJ Monckton JE. Ablation of hippocampal neurogenesis impairs contextual fear conditioning and synaptic plasticity in the dentate gyrus. Proc Natl Acad Sci U S A. (2006) 103:17501–6. doi: 10.1073/pnas.0607207103 PMC1859958 17088541

[48] Meshi D Drew MR Saxe M Ansorge MS David D Santarelli L. Hippocampal neurogenesis is not required for behavioral effects of environmental enrichment. Nat Neurosci. (2006) 9:729–31. doi: 10.1038/nn1696 16648847

[49] Zhang CL Zou Y He W Gage FH Evans RM. A role for adult TLX-positive neural stem cells in learning and behaviour. Nature. (2008) 451:1004–7. doi: 10.1038/nature06562 18235445

[50] Wachowiak M Shipley MT. Coding and synaptic processing of sensory information in the glomerular layer of the olfactory bulb. Semin Cell Dev Biol. (2006) 17:411–23. doi: 10.1016/j.semcdb.2006.04.007 16765614

[51] Mirzadeh Z Merkle FT Soriano-Navarro M Garcia-Verdugo JM Alvarez-Buylla A. Neural stem cells confer unique pinwheel architecture to the ventricular surface in neurogenic regions of the adult brain. Cell Stem Cell. (2008) 3:265–78. doi: 10.1016/j.stem.2008.07.004 PMC2613692 18786414

[52] Shen Q Wang Y Kokovay E Lin G Chuang SM Goderie SK. Adult SVZ stem cells lie in a vascular niche: a quantitative analysis of niche cell-cell interactions. Cell Stem Cell. (2008) 3:289–300. doi: 10.1016/j.stem.2008.07.026 18786416 PMC2747473

[53] Chojnacki AK Mak GK Weiss S. Identity crisis for adult periventricular neural stem cells: subventricular zone astrocytes, ependymal cells or both? Nat Rev Neurosci. (2009) 10:153–63. doi: 10.1038/nrn2571 19153578

[54] Merkle FT Fuentealba LC Sanders TA Magno L Kessaris N Alvarez-Buylla A. Adult neural stem cells in distinct microdomains generate previously unknown interneuron types. Nat Neurosci. (2014) 17:207–14. doi: 10.1038/nn.3610 PMC4100623 24362763

[55] Engler A Rolando C Giachino C Saotome I Erni A Brien C. Notch2 Signaling maintains NSC quiescence in the murine ventricular-subventricular zone. Cell Rep. (2018) 22:992–1002. doi: 10.1016/j.celrep.2017.12.094 29386140

[56] Lim DA Alvarez-Buylla A. The adult ventricular-subventricular zone (V-SVZ) and olfactory bulb (OB) neurogenesis. Cold Spring Harb Perspect Biol. (2016) 8:a018820. doi: 10.1101/cshperspect.a018820 27048191 PMC4852803

[57] Frinchi M Bonomo A Trovato-Salinaro A Condorelli DF Fuxe K Spampinato MG. Fibroblast growth factor-2 and its receptor expression in proliferating precursor cells of the subventricular zone in the adult rat brain. Neurosci Lett. (2008) 447:20–5. doi: 10.1016/j.neulet.2008.09.059 18835325

[58] Gritti A Frölichsthal-Schoeller P Galli R Parati EA Cova L Pagano SF. Epidermal and fibroblast growth factors behave as mitogenic regulators for a single multipotent stem cell-like population from the subventricular region of the adult mouse forebrain. J Neurosci. (1999) 19:3287–97. doi: 10.1523/Ji.19-09-03287.1999 PMC6782245 10212288

[59] Platel JC Lacar B Bordey A. GABA and glutamate signaling: homeostatic control of adult forebrain neurogenesis. J Mol Histol. (2007) 38:303–11. doi: 10.1007/s10735-007-9153-y PMC2556597 17554632

[60] Carleton A Petreanu LT Lansford R Alvarez-Buylla A Lledo PM. Becoming a new neuron in the adult olfactory bulb. Nat Neurosci. (2003) 6:507–18. doi: 10.1038/nn1048 12704391

[61] Whitman MC Greer CA. Adult neurogenesis and the olfactory system. Prog Neurobiol. (2009) 89:162–75. doi: 10.1016/j.pneurobio.2009.07.003 PMC2748178 19615423

[62] Bagley J LaRocca G Jimenez DA Urban NN. Adult neurogenesis and specific replacement of interneuron subtypes in the mouse main olfactory bulb. BMC Neurosci. (2007) 9:8:92. doi: 10.1186/1471-2202-8-92 PMC2238759 17996088

[63] Breton-Provencher V Saghatelyan A. Newborn neurons in the adult olfactory bulb: unique properties for specific odor behavior. Behav Brain Res. (2012) 227:480–9. doi: 10.1016/j.bbr.2011.08.001 21843557

[64] Lazarini F Mouthon MA Gheusi G de Chaumont F Olivo-Marin JC Lamarque S. Cellular and behavioral effects of cranial irradiation of the subventricular zone in adult mice. PloS One. (2009) 4:e701. doi: 10.1371/journal.pone.0007017 PMC2737283 19753118

[65] Breton-Provencher V Lemasson M Peralta MR 3rd Saghatelyan A. Interneurons produced in adulthood are required for the normal functioning of the olfactory bulb network and for the execution of selected olfactory behaviors. J Neurosci. (2009) 29:15245–57. doi: 10.1523/jneurosci.3606-09.2009 PMC6665973 19955377

[66] Sakamoto M Imayoshi I Ohtsuka T Yamaguchi M Mori K Kageyama R. Continuous neurogenesis in the adult forebrain is required for innate olfactory responses. Proc Natl Acad Sci U S A. (2011) 108:8479–84. doi: 10.1073/pnas.1018782108 PMC3100923 21536899

[67] Curtis MA Penney EB Pearson AG van-Roon-Mom WM Butterworth NJ Dragunow M. Increased cell proliferation and neurogenesis in the adult human Huntington’s disease brain. Proc Natl Acad Sci U S A. (2003) 100:9023–7. doi: 10.1073/pnas.1532244100 PMC166431 12853570

[68] Curtis MA Penney EB Pearson J Dragunow M Connor B Faull RL. The distribution of progenitor cells in the subependymal layer of the lateral ventricle in the normal and Huntington’s disease human brain. Neuroscience. (2005) 132:777–88. doi: 10.1016/j.neuroscience.2004.12.051 15837138

[69] Curtis MA Waldvogel HJ Synek B Faull RL. A histochemical and immunohistochemical analysis of the subependymal layer in the normal and Huntington’s disease brain. J Chem Neuroanat. (2005) 30:55–66. doi: 10.1016/j.jchemneu.2005.05.001 16108100

[70] Kopec AM Smith CJ Bilbo SD. Neuro-immune mechanisms regulating social behavior: dopamine as mediator? Trends Neurosci. (2019) 42:337–48. doi: 10.1016/j.tins.2019.02.005 PMC6486862 30890276

[71] Kettenmann H Hanisch UK Noda M Verkhratsky A. Physiology of microglia. Physiol Rev. (2011) 91:461–553. doi: 10.1152/physrev.00011.2010 21527731

[72] Hickman SE Kingery ND Ohsumi TK Borowsky ML Wang LC Means TK. The microglial sensome revealed by direct RNA sequencing. Nat Neurosci. (2013) 16:1896–905. doi: 10.1038/nn.3554 PMC3840123 24162652

[73] Diaz-Aparicio I Paris I Sierra-Torre V Plaza-Zabala A Rodríguez-Iglesias N Márquez-Ropero M. Microglia actively remodel adult hippocampal neurogenesis through the phagocytosis secretome. J Neurosci. (2020) 40:1453–82. doi: 10.1523/jneurosci.0993-19.2019 PMC7044727 31896673

[74] Sierra A Encinas JM Deudero JJ Chancey JH Enikolopov G Overstreet-Wadiche LS. Microglia shape adult hippocampal neurogenesis through apoptosis-coupled phagocytosis. Cell Stem Cell. (2010) 7:483–95. doi: 10.1016/j.stem.2010.08.014 PMC4008496 20887954

[75] Bachstetter AD Morganti JM Jernberg J Schlunk A Mitchell SH Brewster KW. Fractalkine and CX3CR1 regulate hippocampal neurogenesis in adult and aged rats. Neurobiol Aging. (2011) 32:2030–44. doi: 10.1016/j.neurobiolaging.2009.11.022 PMC2889032 20018408

[76] Sellner S Paricio-Montesinos R Spieß A Masuch A Erny D Harsan LA. Microglial CX3CR1 promotes adult neurogenesis by inhibiting Sirt 1/p65 signaling independent of CX3CL1. Acta Neuropathol Commun. (2016) 4:102. doi: 10.1186/s40478-016-0374-8 27639555 PMC5027111

[77] Maggi L Scianni M Branchi I D’Andrea I Lauro C Limatola C. CX(3)CR1 deficiency alters hippocampal-dependent plasticity phenomena blunting the effects of enriched environment. Front Cell Neurosci. (2011) 5:22. doi: 10.1038/nn1928 22025910 PMC3198035

[78] Reshef R Kreisel T Beroukhim Kay D Yirmiya R. Microglia and their CX3CR1 signaling are involved in hippocampal- but not olfactory bulb-related memory and neurogenesis. Brain Behav Immun. (2014) 41:239–50. doi: 10.1016/j.bbi.2014.04.009 24933434

[79] Rogers JT Morganti JM Bachstetter AD Hudson CE Peters MM Grimmig BA. CX3CR1 deficiency leads to impairment of hippocampal cognitive function and synaptic plasticity. J Neurosci. (2011) 31:16241–1650. doi: 10.1523/jneurosci.3667-11.2011 PMC3236509 22072675

[80] Vukovic J Colditz MJ Blackmore DG Ruitenberg MJ Bartlett PF. Microglia modulate hippocampal neural precursor activity in response to exercise and aging. J Neurosci. (2012) 32:6435–43. doi: 10.1523/jneurosci.5925-11.2012 PMC6621117 22573666

[81] Sheridan GK Wdowicz A Pickering M Watters O Halley P O’Sullivan NC. CX3CL1 is up-regulated in the rat hippocampus during memory-associated synaptic plasticity. Front Cell Neurosci. (2014) 8:233. doi: 10.3389/fncel.2014.00233 25161610 PMC4130185

[82] Matarredona ER Talaverón R Pastor AM. Interactions between neural progenitor cells and microglia in the subventricular zone: Physiological implications in the neurogenic niche and after implantation in the injured brain. Front Cell Neurosci. (2018) 12:268. doi: 10.3389/fncel.2018.00268 30177874 PMC6109750

[83] Kyle J Wu M Gourzi S Tsirka SE. Proliferation and differentiation in the adult subventricular zone are not affected by CSF1R inhibition. Front Cell Neurosci. (2019) 13:97. doi: 10.3389/fncel.2019.00097 31001085 PMC6454047

[84] Hurtado-Chong A Yusta-Boyo MJ Vergaño-Vera E Bulfone A de Pablo F Vicario-Abejón C. IGF-I promotes neuronal migration and positioning in the olfactory bulb and the exit of neuroblasts from the subventricular zone. Eur J Neurosci. (2009) 30:742–55. doi: 10.1111/j.1460-9568.2009.06870.x 19712103

[85] Ribeiro Xavier AL Kress BT Goldman SA Lacerda de Menezes JR Nedergaard M. A Distinct population of microglia supports adult neurogenesis in the subventricular zone. J Neurosci. (2015) 35:11848–61. doi: 10.1523/jneurosci.1217-15.2015 PMC4549398 26311768

[86] Möhle L Mattei D Heimesaat MM Bereswill S Fischer A Alutis M. Ly6C(hi) Monocytes provide a link between antibiotic-induced changes in gut microbiota and adult hippocampal neurogenesis. Cell Rep. (2016) 15:1945–56. doi: 10.1016/j.celrep.2016.04.074 27210745

[87] Biswas A Bruder D Wolf SA Jeron A Mack M Heimesaat MM. Ly6C(high) monocytes control cerebral toxoplasmosis. J Immunol. (2015) 194:3223–35. doi: 10.4049/jimmunol.1402037 25710908

[88] Abbas AK Lichtman AH Pillai S. Cellular and Molecular Immunology. 10th Edition. Amsterdam: Elsevier (2022).

[89] Brynskikh A Warren T Zhu J Kipnis J. Adaptive immunity affects learning behavior in mice. Brain Behav Immun. (2008) 22:861–9. doi: 10.1016/j.bbi.2007.12.008 18249087

[90] Huang GJ Smith AL Gray DH Cosgrove C Singer BH Edwards A. A genetic and functional relationship between T cells and cellular proliferation in the adult hippocampus. PloS Biol. (2010) 8:e1000561. doi: 10.1371/journal.pbio.1000561 21179499 PMC3001898

[91] Wolf SA Steiner B Akpinarli A Kammertoens T Nassenstein C Braun A. CD4-positive T lymphocytes provide a neuroimmunological link in the control of adult hippocampal neurogenesis. J Immunol. (2009) 182:3979–84. doi: 10.4049/jimmunol.0801218 19299695

[92] Ziv Y Ron N Butovsky O Landa G Sudai E Greenberg N. Immune cells contribute to the maintenance of neurogenesis and spatial learning abilities in adulthood. Nat Neurosci. (2006) 9:268–75. doi: 10.1038/nn1629 16415867

[93] Liu J Ma Y Tian S Zhang L Zhao M Zhang Y. T cells promote the regeneration of neural precursor cells in the hippocampus of Alzheimer’s disease mice. Neural Regener Res. (2014) 9:1541–7. doi: 10.4103/1673-5374.139481 PMC4192972 25317172

[94] Jeon SG Kim KA Chung H Choi J Song EJ Han SY. Impaired memory in OT-II transgenic mice is associated with decreased adult hippocampal neurogenesis possibly induced by alteration in Th2 cytokine levels. Mol Cells. (2016) 39:603–10. doi: 10.14348/molcells.2016.0072 PMC4990752 27432189

[95] Wang J Xie L Yang C Ren C Zhou K Wang B. Activated regulatory T cell regulates neural stem cell proliferation in the subventricular zone of normal and ischemic mouse brain through interleukin 10. Front Cell Neurosci. (2015) 9:361. doi: 10.3389/fncel.2015.00361 26441532 PMC4568339

[96] Radjavi A Smirnov I Derecki N Kipnis J. Dynamics of the meningeal CD4(+) T-cell repertoire are defined by the cervical lymph nodes and facilitate cognitive task performance in mice. Mol Psychiatry. (2014) 19:531–3. doi: 10.1038/mp.2013.79 PMC3773254 23752249

[97] Herz J Fu Z Kim K Dykstra T Wall M Li H. GABAergic neuronal IL-4R mediates T cell effect on memory. Neuron. (2021) 109:3609–3618.e9. doi: 10.1016/j.neuron.2021.10.022 34793707 PMC9116260

[98] Cushman J Lo J Huang Z Wasserfall C Petitto JM. Neurobehavioral changes resulting from recombinase activation gene 1 deletion. Clin Diagn Lab Immunol. (2003) 10:13–8. doi: 10.1128/cdli.10.1.13-18.2003 PMC145286 12522033

[99] Radjavi A Smirnov I Kipnis J. Brain antigen-reactive CD4+ T cells are sufficient to support learning behavior in mice with limited T cell repertoire. Brain Behav Immun. (2014) 35:58–63. doi: 10.1016/j.bbi.2013.08.013 24012647 PMC3858511

[100] Filella X Molina R Ballesta AM. Estructura y funciones de las citocinas. Med Integr. (2002) 39:63–71.

[101] Callahan MK Ransohoff RM. Analysis of leukocyte extravasation across the blood-brain barrier: conceptual and technical aspects. Curr Allergy Asthma Rep. (2004) 4:65–73. doi: 10.1007/s11882-004-0046-9 14680625

[102] Erickson MA Dohi K Banks WA. Neuroinflammation: a common pathway in CNS diseases as mediated at the blood-brain barrier. Neuroimmunomodulation. (2012) 19:121–30. doi: 10.1159/000330247 PMC3707010 22248728

[103] González-Hernández S Mukouyama YS. Lymphatic vasculature in the central nervous system. Front Cell Dev Biol. (2023) 11:1150775. doi: 10.3389/fcell.2023.1150775 37091974 PMC10119411

[104] Møllgård K Beinlich FRM Kusk P Miyakoshi LM Delle C Plá V. A mesothelium divides the subarachnoid space into functional compartments. Science. (2023) 379:84–8. doi: 10.1126/science.adc8810 36603070

[105] Becher B Spath S Goverman J. Cytokine networks in neuroinflammation. Nat Rev Immunol. (2017) 17:49–59. doi: 10.1038/nri.2016.123 27916979

[106] Vezzani A Viviani B. Neuromodulatory properties of inflammatory cytokines and their impact on neuronal excitability. Neuropharmacology. (2015) 96:70–82. doi: 10.1016/j.neuropharm.2014.10.027 25445483

[107] Dinarello CA. Interleukin-1 in the pathogenesis and treatment of inflammatory diseases. Blood. (2011) 117:3720–32. doi: 10.1182/blood-2010-07-273417 PMC3083294 21304099

[108] Dantzer R. Cytokine, sickness behavior, and depression. Neurol Clin. (2009) 24:441–60. doi: 10.1016/j.ncl.2006.03.003 PMC2909644 16877117

[109] Garlanda C Dinarello CA Mantovani A. The interleukin-1 family: back to the future. Immunity. (2013) 39:1003–18. doi: 10.1016/j.immuni.2013.11.010 PMC3933951 24332029

[110] Cunningham ET Jr Wada E Carter DB Tracey DE Battey JF De Souza EB. *In situ* histochemical localization of type I interleukin-1 receptor messenger RNA in the central nervous system, pituitary, and adrenal gland of the mouse. J Neurosci. (1992) 12:1101–14. doi: 10.1523/jneurosci.12-03-01101.1992 PMC6576039 1532025

[111] Friedman WJ. Cytokines regulate expression of the type 1 interleukin-1 receptor in rat hippocampal neurons and glia. Exp Neurol. (2001) 168:23–31. doi: 10.1006/exnr.2000.7595 11170718

[112] Koo JW Duman RS. IL-1beta is an essential mediator of the antineurogenic and anhedonic effects of stress. Proc Natl Acad Sci USA. (2008) 105:751–6. doi: 10.1073/pnas.0708092105 PMC2206608 18178625

[113] Ryan SM O’Keeffe GW O’Connor C Keeshan K Nolan YM. Negative regulation of TLX by IL-1β correlates with an inhibition of adult hippocampal neural precursor cell proliferation. Brain Behav Immun. (2013) 33:7–13. doi: 10.1016/j.bbi.2013.03.005 23510989

[114] Goshen I Kreisel T Ben-Menachem-Zidon O Licht T Weidenfeld J Ben-Hur T. Brain interleukin-1 mediates chronic stress-induced depression in mice via adrenocortical activation and hippocampal neurogenesis suppression. Mol Psychiatry. (2008) 13:717–28. doi: 10.1038/sj.mp.4002055 17700577

[115] Wu MD Hein AM Moravan MJ Shaftel SS Olschowka JA O’Banion MK. Adult murine hippocampal neurogenesis is inhibited by sustained IL-1β and not rescued by voluntary running. Brain Behav Immun. (2012) 26:292–300. doi: 10.1016/j.bbi.2011.09.012 21983279 PMC3258353

[116] Seguin JA Brennan J Mangano E Hayley S. Proinflammatory cytokines differentially influence adult hippocampal cell proliferation depending upon the route and chronicity of administration. Neuropsychiatr Dis Treat. (2009) 5:5–14. doi: 10.2147/NDT.S4476 19557094 PMC2695223

[117] Kokovay E Wang Y Kusek G Wurster R Lederman P Lowry N. VCAM1 is essential to maintain the structure of the SVZ niche and acts as an environmental sensor to regulate SVZ lineage progression. Cell Stem Cell. (2012) 11:220–30. doi: 10.1016/j.stem.2012.06.016 22862947

[118] Moore AH Wu M Shaftel SS Graham KA O’Banion MK. Sustained expression of interleukin-1beta in mouse hippocampus impairs spatial memory. Neuroscience. (2009) 164:1484–95. doi: 10.1016/j.neuroscience.2009.08.073 PMC2783232 19744544

[119] Hein AM Stasko MR Matousek SB Scott-McKean JJ Maier SF Olschowka JA. Sustained hippocampal IL-1beta overexpression impairs contextual and spatial memory in transgenic mice. Brain Behav Immun. (2010) 24:243–53. doi: 10.1016/j.bbi.2009.10.002 PMC2818290 19825412

[120] Avital A Goshen I Kamsler A Segal M Iverfeldt K Richter-Levin G. Impaired interleukin-1 signaling is associated with deficits in hippocampal memory processes and neural plasticity. Hippocampus. (2003) 13:826–34. doi: 10.1002/hipo.10135 14620878

[121] Bluthé RM Layé S Michaud B Combe C Dantzer R Parnet P. Role of interleukin-1beta and tumour necrosis factor-alpha in lipopolysaccharide-induced sickness behaviour: a study with interleukin-1 type I receptor-deficient mice. Eur J Neurosci. (2000) 12:4447–56. doi: 10.1046/j.1460-9568.2000.01348.x 11122355

[122] Takahashi A Aleyasin H Stavarache MA Li L Cathomas F Parise LF. Neuromodulatory effect of interleukin 1β in the dorsal raphe nucleus on individual differences in aggression. Mol Psychiatry. (2022) 27:2563–79. doi: 10.1038/s41380-021-01110-4 PMC8556414 33931727

[123] Bradley JR. TNF-mediated inflammatory disease. J Pathol. (2008) 214:149–60. doi: 10.1002/path.2287 18161752

[124] Jiang Y Yu M Hu X Han L Yang K Ba H. STAT1 mediates transmembrane TNF-alpha-induced formation of death-inducing signaling complex and apoptotic signaling via TNFR1. Cell Death Differ. (2017) 24:660–71. doi: 10.1038/cdd.2016.162 PMC5384023 28186502

[125] Horiuchi T Mitoma H Harashima S Tsukamoto H Shimoda T. Transmembrane TNF-alpha: structure, function and interaction with anti-TNF agents. Rheumatol (Oxford). (2010) 49:1215–28. doi: 10.1093/rheumatology/keq031 PMC2886310 20194223

[126] Pobezinskaya YL Liu Z. The role of TRADD in death receptor signaling. Cell Cycle. (2012) 11:871–6. doi: 10.4161/cc.11.5.19300 PMC3679287 22333735

[127] Probert L. TNF and its receptors in the CNS: the essential, the desirable and the deleterious effects. Neuroscience. (2015) 302:2–22. doi: 10.1016/j.neuroscience.2015.06.038 26117714

[128] Iosif RE Ekdahl CT Ahlenius H Pronk CJ Bonde S Kokaia Z. Tumor necrosis factor receptor 1 is a negative regulator of progenitor proliferation in adult hippocampal neurogenesis. J Neurosci. (2006) 38:9703–12. doi: 10.1523/jneurosci.2723-06.2006 PMC6674454 16988041

[129] Chen Z Palmer TD. Differential roles of TNFR1 and TNFR2 signaling in adult hippocampal neurogenesis. Brain Behav Immun. (2013) 30:45–53. doi: 10.1016/j.bbi.2013.01.083 23402793 PMC3641155

[130] Matsuda T Murao N Katano Y Juliandi B Kohyama J Akira S. TLR9 signalling in microglia attenuates seizure-induced aberrant neurogenesis in the adult hippocampus. Nat Commun. (2015) 6:6514. doi: 10.1038/ncomms7514 25751136 PMC4366529

[131] Wu JP Kuo JS Liu YL Tzeng SF. Tumor necrosis factor-alpha modulates the proliferation of neural progenitors in the subventricular/ventricular zone of adult rat brain. Neurosci Lett. (2000) 292:203–6. doi: 10.1016/s0304-3940(00)01472-5 11018312

[132] Iosif RE Ahlenius H Ekdahl CT Darsalia V Thored P Jovinge S. Suppression of stroke-induced progenitor proliferation in adult subventricular zone by tumor necrosis factor receptor 1. J Cereb Blood Flow Metab. (2008) 28:1574–87. doi: 10.1038/jcbfm.2008.47 18493257

[133] Wong G Goldshmit Y Turnley AM. Interferon-gamma but not TNF alpha promotes neuronal differentiation and neurite outgrowth of murine adult neural stem cells. Exp Neurol. (2004) 187:171–7. doi: 10.1016/j.expneurol.2004.01.009 15081598

[134] Belenguer G Duart-Abadia P Jordán-Pla A Domingo-Muelas A Blasco-Chamarro L Ferrón SR. Adult neural stem cells are alerted by systemic inflammation through TNF-α receptor signaling. Cell Stem Cell. (2021) 28:285–299.e9. doi: 10.1016/j.stem.2020.10.016 33207218

[135] Widera D Mikenberg I Elvers M Kaltschmidt C Kaltschmidt B. Tumor necrosis factor alpha triggers proliferation of adult neural stem cells via IKK/NF-kappaB signaling. BMC Neurosci. (2006) 7:64. doi: 10.1186/1471-2202-7-64 16987412 PMC1586209

[136] Fransen L Ruysschaert MR van der Heyden J Fiers W. Recombinant tumor necrosis factor: species specificity for a variety of human and murine transformed cell lines. Cell Immunol. (1986) 100:260–7. doi: 10.1016/0008-8749(86)90025-0 3091262

[137] Scherbel U Raghupathi R Nakamura M Saatman KE Trojanowski JQ Neugebauer E. Differential acute and chronic responses of tumor necrosis factor-deficient mice to experimental brain injury. Proc Natl Acad Sci U S A. (1999) 96:8721–6. doi: 10.1073/pnas.96.15.8721 PMC17583 10411942

[138] Baune BT Wiede F Braun A Golledge J Arolt V Koerner H. Cognitive dysfunction in mice deficient for TNF- and its receptors. Am J Med Genet B Neuropsychiatr Genet. (2008) 147B:1056–64. doi: 10.1002/ajmg.b.30712 18286589

[139] Mygind L Bergh MS Tejsi V Vaitheeswaran R Lambertsen KL Finsen B. Tumor necrosis factor (TNF) is required for spatial learning and memory in male mice under physiological, but not immune-challenged conditions. Cells. (2021) 10:608. doi: 10.3390/cells10030608 33803476 PMC8002217

[140] Patel A Siegel A Zalcman SS. Lack of aggression and anxiolytic-like behavior in TNF receptor (TNF-R1 and TNF-R2) deficient mice. Brain Behav Immun. (2010) 24:1276–80. doi: 10.1016/j.bbi.2010.05.005 PMC3119927 20685290

[141] Hirano T Yasukawa K Harada H Taga T Watanabe Y Matsuda T. Complementary DNA for a novel human interleukin (BSF-2) that induces B lymphocytes to produce immunoglobulin. Nature. (1986) 324:73–6. doi: 10.1038/324073a0 3491322

[142] Boulanger MJ Chow DC Brevnova EE Garcia KC. Hexameric structure and assembly of the interleukin-6/IL-6 alpha-receptor/gp130 complex. Science. (2003) 300:2101–4. doi: 10.1126/science.1083901 12829785

[143] Hunter CA Jones SA. IL-6 as a keystone cytokine in health and disease. Nat Immunol. (2015) 16:448–57. doi: 10.1038/ni.3153 25898198

[144] Jones SA Jenkins BJ. Recent insights into targeting the IL-6 cytokine family in inflammatory diseases and cancer. Nat Rev Immunol. (2018) 18:773–89. doi: 10.1038/s41577-018-0066-7 30254251

[145] Riethmueller S Somasundaram P Ehlers JC Hung CW Flynn CM Lokau J. Proteolytic origin of the soluble human IL-6R *in vivo* and a decisive role of N-glycosylation. PloS Biol. (2017) 15:e2000080. doi: 10.1371/journal.pbio.2000080 28060820 PMC5218472

[146] Müllberg J Althoff K Jostock T Rose-John S. The importance of shedding of membrane proteins for cytokine biology. Eur Cytokine Netw. (2000) 11:27–38. 10705296

[147] Rose-John S. Local and systemic effects of interleukin-6 (IL-6) in inflammation and cancer. FEBS Lett. (2022) 596(5):557–66. doi: 10.1002/1873-3468.14220 34738234

[148] Scheller J Chalaris A Garbers C Rose-John S. ADAM17: a molecular switch to control inflammation and tissue regeneration. Trends Immunol. (2011) 32:380–7. doi: 10.1016/j.it.2011.05.005 21752713

[149] Erta M Quintana A Hidalgo J. Interleukin-6, a major cytokine in the central nervous system. Int J Biol Sci. (2012) 8(9):1254–66. doi: 10.7150/ijbs.4679 PMC3491449 23136554

[150] McPherson CA Aoyama M Harry GJ. Interleukin (IL)-1 and IL-6 regulation of neural progenitor cell proliferation with hippocampal injury: differential regulatory pathways in the subgranular zone (SGZ) of the adolescent and mature mouse brain. Brain Behav Immun. (2011) 25:850–62. doi: 10.1016/j.bbi.2010.09.003 PMC3033445 20833246

[151] Storer MA Gallagher D Fatt MP Simonetta JV Kaplan DR Miller FD. Interleukin-6 regulates adult neural stem cell numbers during normal and abnormal Post-natal Development. Stem Cell Rep. (2018) 10:1464–80. doi: 10.1016/j.stemcr.2018.03.008 PMC5995693 29628394

[152] Monje ML Toda H Palmer TD. Inflammatory blockade restores adult hippocampal neurogenesis. Science. (2003) 302:1760–5. doi: 10.1126/science.1088417 14615545

[153] Zonis S Ljubimov VA Mahgerefteh M Pechnick RN Wawrowsky K Chesnokova V. p21Cip restrains hippocampal neurogenesis and protects neuronal progenitors from apoptosis during acute systemic inflammation. Hippocampus. (2013) 23:1383–94. doi: 10.1002/hipo.22192 PMC4170945 23966332

[154] Oh J McCloskey MA Blong CC Bendickson L Nilsen-Hamilton M Sakaguchi DS. Astrocyte-derived interleukin-6 promotes specific neuronal differentiation of neural progenitor cells from adult hippocampus. J Neurosci Res. (2010) 88:2798–809. doi: 10.1002/jnr.22447 PMC2981827 20568291

[155] Vallières L Campbell IL Gage FH Sawchenko PE. Reduced hippocampal neurogenesis in adult transgenic mice with chronic astrocytic production of interleukin-6. J Neurosci. (2002) 22:486–92. doi: 10.1523/jneurosci.22-02-00486.2002 PMC6758670 11784794

[156] Bowen KK Dempsey RJ Vemuganti R. Adult interleukin-6 knockout mice show compromised neurogenesis. Neuroreport. (2011) 22:126–30. doi: 10.1097/WNR.0b013e3283430a44 21266900

[157] Braida D Sacerdote P Panerai AE Bianchi M Aloisi AM Iosuè S. Cognitive function in young and adult IL (interleukin)-6 deficient mice. Behav Brain Res. (2004) 153:423–9. doi: 10.1016/j.bbr.2003.12.018 15265638

[158] Baier PC May U Scheller J Rose-John S Schiffelholz T. Impaired hippocampus-dependent and -independent learning in IL-6 deficient mice. Behav Brain Res. (2009) 8:192–6. doi: 10.1016/j.bbr.2009.01.013 19378383

[159] Bialuk I Jakubów P Winnicka MM. Significance of IL-6 deficiency in recognition memory in young adult and aged mice. Behav Genet. (2019) 49:415–23. doi: 10.1007/s10519-019-09959-6 PMC6554246 31129771

[160] Hryniewicz A Bialuk I Kamiński KA Winnicka MM. Impairment of recognition memory in interleukin-6 knock-out mice. Eur J Pharmacol. (2007) 577:219–20. doi: 10.1016/j.ejphar.2007.08.046 17920057

[161] Chesworth R Gamage R Ullah F Sonego S Millington C Fernandez A. Spatial memory and microglia activation in a mouse model of chronic neuroinflammation and the anti-inflammatory effects of apigenin. Front Neurosci. (2021) 15:699329. doi: 10.3389/fnins.2021.699329 34393713 PMC8363202

[162] Heyser CJ Masliah E Samimi A Campbell IL Gold LH. Progressive decline in avoidance learning paralleled by inflammatory neurodegeneration in transgenic mice expressing interleukin 6 in the brain. Proc Natl Acad Sci U S A. (1997) 94:1500–5. doi: 10.1073/pnas.94.4.1500 PMC19820 9037082

[163] Wei H Chadman KK McCloskey DP Sheikh AM Malik M Brown WT. Brain IL-6 elevation causes neuronal circuitry imbalances and mediates autism-like behaviors. Biochim Biophys Acta. (2012) 1822:831–42. doi: 10.1016/j.bbadis.2012.01.011 22326556

[164] Bialuk I Taranta A Winnicka MM. IL-6 deficiency alters spatial memory in 4- and 24-month-old mice. Neurobiol Learn Mem. (2018) 155:21–9. doi: 10.1016/j.nlm.2018.06.006 29908286

[165] Bialuk I Winnicka MM. Facilitatory Effect of IL-6 Deficiency on long-term spatial memory in young adult mice. Behav Genet. (2018) 48:236–46. doi: 10.1007/s10519-018-9896-0 29619678

[166] Yang Y Lundqvist A. Immunomodulatory effects of IL-2 and IL-15; implications for cancer immunotherapy. Cancers (Basel). (2020) 30:12: 3586. doi: 10.3390/cancers12123586 PMC7761238 33266177

[167] Lee H Park SH Shin EC. IL-15 in T-cell responses and immunopathogenesis. Immune Netw. (2024) 24:e11. doi: 10.4110/in.2024.24.e11 38455459 PMC10917573

[168] Gómez-Nicola D Valle-Argos B Pallas-Bazarra N Nieto-Sampedro M. Interleukin-15 regulates proliferation and self-renewal of adult neural stem cells. Mol Biol Cell. (2011) 22:1960–70. doi: 10.1091/mbc.E11-01-0053 PMC3113763 21508317

[169] Umehara T Udagawa J Takamura K Kimura M Ishimitsu R Kiyono H. Role of interleukin-15 in the development of mouse olfactory nerve. Congenit Anom (Kyoto). (2009) 49:253–7. doi: 10.1111/j.1741-4520.2009.00246.x 20021484

[170] He Y Hsuchou H Wu X Kastin AJ Khan RS Pistell PJ. Interleukin-15 receptor is essential to facilitate GABA transmission and hippocampal-dependent memory. J Neurosci. (2010) 30:4725–34. doi: 10.1523/jneurosci.6160-09.2010 PMC2862729 20357123

[171] Dafny N Prieto-Gomez B Dong WQ Reyes-Vazquez C. Interferon modulates neuronal activity recorded from the hypothalamus, thalamus, hippocampus, amygdala and the somatosensory cortex. Brain Res. (1996) 734:269–74. doi: 10.1016/0006-8993(96)00650-6 8896834

[172] Paul S Ricour C Sommereyns C Sorgeloos F Michiels T. Type I interferon response in the central nervous system. Biochimie. (2007) 89:770–8. doi: 10.1016/j.biochi.2007.02.009 17408841

[173] Zheng LS Hitoshi S Kaneko N Takao K Miyakawa T Tanaka Y. Mechanisms for interferon-α-induced depression and neural stem cell dysfunction. Stem Cell Rep. (2014) 3:73–84. doi: 10.1016/j.stemcr.2014.05.015 PMC4110771 25068123

[174] Kaneko N Kudo K Mabuchi T Takemoto K Fujimaki K Wati H. Suppression of cell proliferation by interferon-alpha through interleukin-1 production in adult rat dentate gyrus. Neuropsychopharmacology. (2006) 31:2619–26. doi: 10.1038/sj.npp.1301137 16823390

[175] Zheng LS Kaneko N Sawamoto K. Minocycline treatment ameliorates interferon-alpha-induced neurogenic defects and depression-like behaviors in mice. Front Cell Neurosci. (2015) 9:5. doi: 10.3389/fncel.2015.00005 25674053 PMC4309184

[176] Lum M Croze E Wagner C McLenachan S Mitrovic B Turnley AM. Inhibition of neurosphere proliferation by IFN gamma but not IFN beta is coupled to neuronal differentiation. J Neuroimmunol. (2009) 206:32–8. doi: 10.1016/j.jneuroim.2008.10.009 19027965

[177] Hosseini S Michaelsen-Preusse K Grigoryan G Chhatbar C Kalinke U Korte M. Type I Interferon receptor signaling in astrocytes regulates hippocampal synaptic plasticity and cognitive function of the healthy CNS. Cell Rep. (2020) 31:107666. doi: 10.1016/j.celrep.2020.107666 32433975

[178] Becher B Tugues S Greter M. GM-CSF: from growth factor to central mediator of tissue inflammation. Immunity. (2016) 45:963–73. doi: 10.1016/j.immuni.2016.10.026 27851925

[179] Krüger C Laage R Pitzer C Schäbitz WR Schneider A. The hematopoietic factor GM-CSF (granulocyte-macrophage colony-stimulating factor) promotes neuronal differentiation of adult neural stem cells *in vitro* . BMC Neurosci. (2007) 8:88. doi: 10.1186/1471-2202-8-88 17953750 PMC2233634

[180] Krieger M Both M Kranig SA Pitzer C Klugmann M Vogt G. The hematopoietic cytokine granulocyte-macrophage colony stimulating factor is important for cognitive functions. Sci Rep. (2012) 2:697. doi: 10.1038/srep00697 23019518 PMC3458247

[181] Ishiguro M Okada A Asai K Kojima K Okada H. Stimulation of neuronal cells by culture supernatant of T lymphocytes triggered by anti-CD3 mAb followed by propagation in the presence of interleukin-2. Microbiol Immunol. (2016) 60:47–55. doi: 10.1111/1348-0421.12346 26616436

[182] Kiyota T Machhi J Lu Y Dyavarshetty B Nemati M Yokoyama I. Granulocyte-macrophage colony-stimulating factor neuroprotective activities in Alzheimer’s disease mice. J Neuroimmunol. (2018) 319:80–92. doi: 10.1016/j.jneuroim.2018.03.009 29573847 PMC5916331

[183] Ahmed MM Wang AC Elos M Chial HJ Sillau S Solano DA. The innate immune system stimulating cytokine GM-CSF improves learning/memory and interneuron and astrocyte brain pathology in Dp16 Down syndrome mice and improves learning/memory in wild-type mice. Neurobiol Dis. (2022) 168:105694. doi: 10.1016/j.nbd.2022.105694 35307513 PMC9045510

[184] Dogan RN Elhofy A Karpus WJ. Production of CCL2 by central nervous system cells regulates development of murine experimental autoimmune encephalomyelitis through the recruitment of TNF- and iNOS-expressing macrophages and myeloid dendritic cells. J Immunol. (2008) 180:7376–84. doi: 10.4049/jimmunol.180.11.7376 18490737

[185] Banisadr G Quéraud-Lesaux F Boutterin MC Pélaprat D Zalc B Rostène W. Distribution, cellular localization and functional role of CCR2 chemokine receptors in adult rat brain. J Neurochem. (2002) 81:257–69. doi: 10.1046/j.1471-4159.2002.00809.x 12064472

[186] Gordon RJ Mehrabi NF Maucksch C Connor B. Chemokines influence the migration and fate of neural precursor cells from the young adult and middle-aged rat subventricular zone. Exp Neurol. (2012) 233:587–94. doi: 10.1016/j.expneurol.2011.11.029 22155482

[187] Xu JH Long L Tang YC Zhang JT Hut HT Tang FR. CCR3, CCR2A and macrophage inflammatory protein (MIP)-1a, monocyte chemotactic protein-1 (MCP-1) in the mouse hippocampus during and after pilocarpine-induced status epilepticus (PISE). Neuropathol Appl Neurobiol. (2009) 35:496–514. doi: 10.1111/j.1365-2990.2009.01022.x 19490431

[188] Ji JF He BP Dheen ST Tay SS. Expression of chemokine receptors CXCR4, CCR2, CCR5 and CX3CR1 in neural progenitor cells isolated from the subventricular zone of the adult rat brain. Neurosci Lett. (2004) 355:236–40. doi: 10.1016/j.neulet.2003.11.024 14732474

[189] Liu XS Zhang ZG Zhang RL Gregg SR Wang L Yier T. Chemokine ligand 2 (CCL2) induces migration and differentiation of subventricular zone cells after stroke. J Neurosci Res. (2007) 85:2120–5. doi: 10.1002/jnr.21359 17510981

[190] Marciniak E Faivre E Dutar P Alves Pires C Demeyer D Caillierez R. The Chemokine MIP-1α/CCL3 impairs mouse hippocampal synaptic transmission, plasticity and memory. Sci Rep. (2015) 5:15862. doi: 10.1038/srep15862 26511387 PMC4625372

[191] Villeda SA Luo J Mosher KI Zou B Britschgi M Bieri G. The aging systemic milieu negatively regulates neurogenesis and cognitive function. Nature. (2011) 477:90–4. doi: 10.1038/nature10357 PMC3170097 21886162

[192] Stumm RK Rummel J Junker V Culmsee C Pfeiffer M Krieglstein J. A dual role for the SDF-1/CXCR4 chemokine receptor system in adult brain: isoform-selective regulation of SDF-1 expression modulates CXCR4-dependent neuronal plasticity and cerebral leukocyte recruitment after focal ischemia. J Neurosci. (2002) 22:5865–78. doi: 10.1523/jneurosci.22-14-05865.2002 PMC6757949 12122049

[193] Sowa JE Tokarski K. Cellular, synaptic, and network effects of chemokines in the central nervous system and their implications to behavior. Pharmacol Rep. (2021) 73:1595–625. doi: 10.1007/s43440-021-00323-2 PMC8599319 34498203

[194] Abe P Wüst HM Arnold SJ van de Pavert SA Stumm R. CXCL12-mediated feedback from granule neurons regulates generation and positioning of new neurons in the dentate gyrus. Glia. (2018) 66:1566–76. doi: 10.1002/glia.23324 29537098

[195] Trousse F Jemli A Silhol M Garrido E Crouzier L Naert G. Knockdown of the CXCL12/CXCR7 chemokine pathway results in learning deficits and neural progenitor maturation impairment in mice. Brain Behav Immun. (2019) 80:697–710. doi: 10.1016/j.bbi.2019.05.019 31100368

[196] Anonymous. Interferon nomenclature. Nature. (1980) 286:110. doi: 10.1038/286110a0 6157097

[197] Wheelock EF. Interferon-like virus-inhibitor induced in human leukocytes by phytohemagglutinin. Science. (1965) 16:149:310–311. doi: 10.1126/science.149.3681.310 17533668

[198] Billiau A Matthys P. Interferon-gamma: a historical perspective. Cytokine Growth Factor Rev. (2009) 20:97–113. doi: 10.1016/j.cytogfr.2009.02.004 19268625

[199] Popko B Corbin JG Baerwald KD Dupree J Garcia AM. The effects of interferon-gamma on the central nervous system. Mol Neurobiol. (1997) 14:19–35. doi: 10.1007/BF02740619 9170099 PMC7091409

[200] Baron R Nemirovsky A Harpaz I Cohen H Owens T Monsonego A. IFN-gamma enhances neurogenesis in wild-type mice and in a mouse model of Alzheimer’s disease. FASEB J. (2008) 22:2843–52. doi: 10.1096/fj.08-105866 18390924

[201] Campos AC Vaz GN Saito VM Teixeira AL. Further evidence for the role of interferon-gamma on anxiety- and depressive-like behaviors: involvement of hippocampal neurogenesis and NGF production. Neurosci Lett. (2014) 578:100–5. doi: 10.1016/j.neulet.2014.06.039 24993299

[202] Monteiro S Ferreira FM Pinto V Roque S Morais M de-Sá-Calçada D. Absence of IFNγ promotes hippocampal plasticity and enhances cognitive performance. Transl Psychiatry. (2016) 6:e707. doi: 10.1038/tp.2015.194 26731444 PMC5073154

[203] Zhang J He H Qiao Y Zhou T He H Yi S. Priming of microglia with IFN-γ impairs adult hippocampal neurogenesis and leads to depression-like behaviors and cognitive defects. Glia. (2020) 68:2674–92:.32652855. doi: 10.1002/glia.23878 32652855

[204] Cossetti C Iraci N Mercer TR Leonardi T Alpi E Drago D. Extracellular vesicles from neural stem cells transfer IFN-γ via Ifngr1 to activate Stat1 signaling in target cells. Mol Cell. (2014) 56:193–204. doi: 10.1016/j.molcel.2014.08.020 25242146 PMC4578249

[205] Turbic A Leong SY Turnley AM. Chemokines and inflammatory mediators interact to regulate adult murine neural precursor cell proliferation, survival and differentiation. PloS One. (2011) 6:e25406. doi: 10.1371/journal.pone.0025406 21966521 PMC3179517

[206] Li L Walker TL Zhang Y Mackay EW Bartlett PF. Endogenous interferon gamma directly regulates neural precursors in the non-inflammatory brain. J Neurosci. (2010) 30:9038–50. doi: 10.1523/jneurosci.5691-09.2010 PMC6632462 20610738

[207] Pereira L Medina R Baena M Planas AM Pozas E. IFN gamma regulates proliferation and neuronal differentiation by STAT1 in adult SVZ niche. Front Cell Neurosci. (2015) 9:270. doi: 10.3389/fncel.2015.00270 26217191 PMC4499753

[208] Filiano AJ Xu Y Tustison NJ Marsh RL Baker W Smirnov I. Unexpected role of interferon-γ in regulating neuronal connectivity and social behaviour. Nature. (2016) 535:425–9. doi: 10.1038/nature18626 PMC4961620 27409813

[209] Keegan AD Leonard WJ Zhu J. Recent advances in understanding the role of IL-4 signaling. Fac Rev. (2021) 10:71. doi: 10.12703/r/10-71 34557875 PMC8442009

[210] Hanuscheck N Thalman C Domingues M Schmaul S Muthuraman M Hetsch F. Interleukin-4 receptor signaling modulates neuronal network activity. J Exp Med. (2022) 219:e20211887. doi: 10.1084/jem.20211887 35587822 PMC9123307

[211] Mashkaryan V Siddiqui T Popova S Cosacak MI Bhattarai P Brandt K. Type 1 Interleukin-4 signaling obliterates mouse astroglia *in vivo* but Not *in vitro* . Front Cell Dev Biol. (2020) 8:114. doi: 10.3389/fcell.2020.00114 32181251 PMC7057913

[212] Zhang J Rong P Zhang L He H Zhou T Fan Y. IL4-driven microglia modulate stress resilience through BDNF-dependent neurogenesis. Sci Adv. (2021) 7:eabb9888. doi: 10.1126/sciadv.abb9888 33731342 PMC7968840

[213] Guan Y Jiang Z Ciric B Rostami AM Zhang GX. Upregulation of chemokine receptor expression by IL-10/IL-4 in adult neural stem cells. Exp Mol Pathol. (2008) 85:232–6. doi: 10.1016/j.yexmp.2008.07.003 18775694

[214] Brombacher TM Nono JK De Gouveia KS Makena N Darby M Womersley J. IL-13-mediated regulation of learning and memory. J Immunol. (2017) 198:2681–8. doi: 10.4049/jimmunol.1601546 28202615

[215] Brombacher TM Ajonijebu DC Scibiorek M Berkiks I Moses BO Mpotje T. IL-4Rα deletion disrupts psychomotor performance and reference memory in mice while sparing behavioural phenotype associated with spatial learning. Brain Behav Immun. (2021) 92:157–64. doi: 10.1016/j.bbi.2020.12.003 PMC7909383 33301870

[216] Derecki NC Cardani AN Yang CH Quinnies KM Crihfield A Lynch KR. Regulation of learning and memory by meningeal immunity: a key role for IL-4. J Exp Med. (2010) 207:1067–980. doi: 10.1084/jem.20091419 PMC2867291 20439540

[217] Moon ML Joesting JJ Blevins NA Lawson MA Gainey SJ Towers AE. IL-4 knockout mice display anxiety-like behavior. Behav Genet. (2015) 45:451–60. doi: 10.1007/s10519-015-9714-x PMC4459943 25772794

[218] Liu Q Xin W He P Turner D Yin J Gan Y. Interleukin-17 inhibits adult hippocampal neurogenesis. Sci Rep. (2014) 4:7554. doi: 10.1038/srep07554 25523081 PMC4271266

[219] Tfilin M Turgeman G. Interleukine-17 administration modulates adult hippocampal neurogenesis and improves spatial learning in mice. J Mol Neurosci. (2019) 69:254–63. doi: 10.1007/s12031-019-01354-4 31254254

[220] Willinger Y Turgeman G. Interleukin-17 modulates neurogenesis and behavior following exposure to trauma in mice. Cells. (2022) 20:11:343. doi: 10.3390/cells11030343 PMC8834196 35159158

[221] Ribeiro M Brigas HC Temido-Ferreira M Pousinha PA Regen T Santa C. Meningeal γδ T cell-derived IL-17 controls synaptic plasticity and short-term memory. Sci Immunol. (2019) 4:easy 5199. doi: 10.1126/sciimmunol.aay5199 PMC6894940 31604844

[222] Floss DM Schröder J Franke M Scheller J. Insights into IL-23 biology: From structure to function. Cytokine Growth Factor Rev. (2015) 26:569–78. doi: 10.1016/j.cytogfr.2015.07.005 26195433

[223] Araujo DM Lapchak PA Collier B Quirion R. Localization of interleukin-2 immunoreactivity and interleukin-2 receptors in the rat brain: interaction with the cholinergic system. Brain Res. (1989) 498:257–66. doi: 10.1016/0006-8993(89)91104-9 2790482

[224] Lapchak PA Araujo DM Quirion R Beaudet A. Immunoautoradiographic localization of interleukin 2-like immunoreactivity and interleukin 2 receptors (Tac antigen-like immunoreactivity) in the rat brain. Neuroscience. (1991) 44:173–84. doi: 10.1016/0306-4522(91)90259-q 1770995

[225] Beck RD Jr Wasserfall C Ha GK Cushman JD Huang Z Atkinson MA. Changes in hippocampal IL-15, related cytokines, and neurogenesis in IL-2 deficient mice. Brain Res. (2005) 1041:223–30. doi: 10.1016/j.brainres.2005.02.010 15829231

[226] Petitto JM McNamara RK Gendreau PL Huang Z Jackson AJ. Impaired learning and memory and altered hippocampal neurodevelopment resulting from interleukin-2 gene deletion. J Neurosci Res. (1999) 56:441–6. doi: 10.1002/(SICI)1097-4547(19990515)56:4<441::AID-JNR11>3.0.CO;2-G 10340751

[227] Petitto JM Huang Z Hartemink DA Beck R Jr. IL-2/15 receptor-beta gene deletion alters neurobehavioral performance. Brain Res. (2002) 929:218–25. doi: 10.1016/s0006-8993(01)03393-5 11864627

[228] Wu X Kastin AJ Hsuchou H Pan W. The effects of IL2R gamma knockout on depression and contextual memory. Behav Brain Res. (2010) 213:319–22. doi: 10.1016/j.bbr.2010.04.046 PMC2907353 20438766

[229] Fiorentino DF Bond MW Mosmann TR. Two types of mouse T helper cell. IV. Th2 clones secrete a factor that inhibits cytokine production by Th1 clones. J Exp Med. (1989) 170:2081–95. doi: 10.1084/jem.170.6.2081 PMC2189521 2531194

[230] Gabryšová L Howes A Saraiva M O’Garra A. The regulation of IL-10 expression. Curr Top Microbiol Immunol. (2014) 380:157–90. 10.1007/978-3-662-43492-5_8 25004818

[231] Howes A Gabryšová L O’Garra A. Role of IL-10 and the IL-10 receptor in immune responses. in: Reference Module in Biomedical Sciences. 3rd edition. London: Elsevier (2014) p. 1–11.

[232] Lim SH Park E You B Jung Y Park AR Park SG. Neuronal synapse formation induced by microglia and interleukin 10. PloS One. (2013) 8:e81218. doi: 10.1371/journal.pone.0081218 24278397 PMC3838367

[233] Segev-Amzaleg N Trudler D Frenkel D. Preconditioning to mild oxidative stress mediates astroglial neuroprotection in an IL-10-dependent manner. Brain Behav Immun. (2013) 30:176–85. doi: 10.1016/j.bbi.2012.12.016 23313057

[234] Sanchez-Molina P Almolda B Giménez-Llort L González B Castellano B. Chronic IL-10 overproduction disrupts microglia-neuron dialogue similar to aging, resulting in impaired hippocampal neurogenesis and spatial memory. Brain Behav Immun. (2022) 101:231–45. doi: 10.1016/j.bbi.2021.12.026 34990747

[235] Perez-Asensio FJ Perpiñá U Planas AM Pozas E. Interleukin-10 regulates progenitor differentiation and modulates neurogenesis in adult brain. J Cell Sci. (2013) 126:4208–19. doi: 10.1242/jcs.127803 23843621

[236] Cebrian-Silla A Nascimento MA Redmond SA Mansky B Wu D Obernier K. Single-cell analysis of the ventricular-subventricular zone reveals signatures of dorsal and ventral adult neurogenesis. Elife. (2021) 10:e67436. doi: 10.7554/eLife.67436 34259628 PMC8443251

[237] Zhang HY Wang Y He Y Wang T Huang XH Zhao CM. A1 astrocytes contribute to murine depression-like behavior and cognitive dysfunction, which can be alleviated by IL-10 or fluorocitrate treatment. J Neuroinflamm. (2020) 17:200. doi: 10.1186/s12974-020-01871-9 PMC7331266 32611425

[238] Larson C Oronsky B Carter CA Oronsky A Knox SJ Sher D. TGF-beta: a master immune regulator. Expert Opin Ther Targets. (2020) 24:427–38. doi: 10.1080/14728222.2020.1744568 32228232

[239] Roberts AB Anzano MA Wakefield LM Roche NS Stern DF Sporn MB. Type beta transforming growth factor: a bifunctional regulator of cellular growth. Proc Natl Acad Sci U S A. (1985) 82:119–23. doi: 10.1073/pnas.82.1.119 PMC396983 3871521

[240] Bedolla A Wegman E Weed M Stevens MK Ware K Paranjpe A. Adult microglial TGFβ1 is required for microglia homeostasis via an autocrine mechanism to maintain cognitive function in mice. Nat Commun. (2024) 15:5306. doi: 10.1038/s41467-024-49596-0 38906887 PMC11192737

[241] Böttner M Unsicker K Suter-Crazzolara C. Expression of TGF-beta type II receptor mRNA in the CNS. Neuroreport. (1996) 7:2903–7. doi: 10.1097/00001756-199611250-00019 9116206

[242] Wachs FP Winner B Couillard-Despres S Schiller T Aigner R Winkler J. Transforming growth factor-beta1 is a negative modulator of adult neurogenesis. J Neuropathol Exp Neurol. (2006) 65:358–70. doi: 10.1097/01.jnen.0000218444.53405.f0 16691117

[243] Buckwalter MS Yamane M Coleman BS Ormerod BK Chin JT Palmer T. Chronically increased transforming growth factor-beta1 strongly inhibits hippocampal neurogenesis in aged mice. Am J Pathol. (2006) 169:154–64. doi: 10.2353/ajpath.2006.051272 PMC1698757 16816369

[244] Kandasamy M Lehner B Kraus S Sander PR Marschallinger J Rivera FJ. TGF-beta signalling in the adult neurogenic niche promotes stem cell quiescence as well as generation of new neurons. J Cell Mol Med. (2014) 18:1444–59. doi: 10.1111/jcmm.12298 PMC4124027 24779367

[245] Mathieu P Piantanida AP Pitossi F. Chronic expression of transforming growth factor-beta enhances adult neurogenesis. Neuroimmunomodulation. (2010) 17:200–2001. doi: 10.1159/000258723 20134202

[246] Colak D Mori T Brill MS Pfeifer A Falk S Deng C. Adult neurogenesis requires Smad4-mediated bone morphogenetic protein signaling in stem cells. J Neurosci. (2008) 28:434–46. doi: 10.1523/jneurosci.4374-07.2008 PMC6670509 18184786

[247] Shih TW Lee LJ Chang HC Lin HW Chang MS. An important role of PHRF1 in dendritic architecture and memory formation by modulating TGF-β signaling. Sci Rep. (2020) 10:10857. doi: 10.1038/s41598-020-67675-2 32616804 PMC7331665

[248] Depino AM LucChina L Pitossi F. Early and adult hippocampal TGF-β1 overexpression have opposite effects on behavior. Brain Behav Immun. (2011) 25:1582–91. doi: 10.1016/j.bbi.2011.05.007 21640817

[249] Dantzer R O’Connor JC Freund GG Johnson RW Kelley KW. From inflammation to sickness and depression: when the immune system subjugates the brain. Nat Rev Neurosci. (2008) 9:46–56. doi: 10.1038/nrn2297 18073775 PMC2919277

