# Supplementary material for: Controversies and insights into cytokine regulation of neurogenesis and behavior in adult rodents
Source: Front Immunol. 2025 Apr 25;16:1550660. doi: 10.3389/fimmu.2025.1550660 (PMC12061686; doi:10.3389/fimmu.2025.1550660)
Supplement: Supplementary file 1 [file Table1.docx]

**Appendix. Glossary**

- **Achaete-scute homolog 1 (Ascl1) (Mash1)**: bHLH transcription factor essential in the neuronal commitment, differentiation and in the generation of olfactory and autonomic neurons.
- **Barnes Maze Test (BMT)**: dry-land based rodent behavioral paradigm used to measure spatial learning and memory developed by Dr. Carol Barnes.
- **Brain-Blood Barrier (BBB)**: Semipermeable membrane located between the bloodstream and the interstitial space of the brain, which filters the passage of molecules, ions and cells from the blood of cerebral blood vessels.
- **Brain-Derived Neurotrophic Factor (BDNF)**: protein derived from CD4+ cells crucial in neuronal survival and growth, neurotransmitter modulation, neurogenesis, memory, and learning.
- **Bromodeoxyuridine (BrdU)**: Synthetic biomarker analogue of thymidine that is incorporated into the DNA of dividing cells during the S phase of the cell cycle. It is used to date cell births and determine the level of control of cell proliferation and survival.
- **Monocyte chemoattractant protein-1 (CCL2)**: chemokine member of the CC chemokine family that has chemoattractant activity for monocytes, T cells, mast cells, and basophils.
- **Eotaxin (CLL11)**: chemokine with an important role in allergic conditions.
- **Eotaxin-3 (CCL26)**: chemokines involved in the recruitment of eosinophils into tissues and mainly activate CCR3.
- **Macrophage Inflammatory Protein-1 Alpha (CCL3)**: chemotactic chemokine secreted by macrophages which recruit inflammatory cells, wound healing, inhibition of stem cells, and maintaining effector immune response.
- **Central Nervous System (CNS)**: anatomical set made up of the brain and the spinal cord, which is responsible for processing the information received from the body's sensory organs and coordinating organic responses.
- Peripheral Nervous System (PNS)
- **Chemokines**: family of small, secreted proteins that stimulate the migration of cells, most notably leukocytes.
- **Cytokines**: a broad and diverse group of small proteins that play a crucial role in cell signaling and are involved in communication between cells of the immune system and are key regulatorse of various physiological processes.
- **Doublecortin (DCX)**: microtubule protein expressed almost exclusively in immature neurons, this protein binds to microtubules in migrating cells and as a result promotes the movement of these cells working like a neural migration biomarker.
- **Dentate gyrus (DG)**: is a simple cortical region of the hippocampus integrated principally by granule cells and it is associated with the formation of our episodic memory as well as the exploration of new environments.
- **Gamma-amino-butiric acid (GABA)**:amino acid that serves as the primary inhibitory neurotransmitter in the brain and spinal cord.
- **Glial fibrillary acidic protein (GFAP)**: intermediate filament protein present in astrocytes of the central nervous system that works like a biomarker of astrocytes.
- **Granule neurons (GN)**: are the smallest neurons of the brain and the principal neurons of the dentate gyrus.
- **Granulocyte colony stimulating factor (G-CSF)**: is a glycoprotein that stimulates the bone marrow to produce granulocytes and stem cells and release them into the bloodstream.
- **Growth factor-beta (TGF-β)**: secreted polypeptide factors synthesized by lymphocytes, macrophages and dendritic cells; that regulate cell proliferation, differentiation and apoptosis.
- **Chemokine growth-regulated protein alpha (GRO-α)**: gene which transcript that increase in abundance with age in organs, inter alia in epithelial skin cells, chronically expressed ti is associated with tissue damage, angiogenesis, and tumorigenesis can follow.
- **Interferon alfa (IFN-α)**: immunomodulatory antiviral cytokine of the type I IFN innate immune response of most cell types produced mainly by plasmacytoid dendritic cells.
- **Interferon beta (IFN-β)**: immunomodulatory cytokine mainly by macrophages and plasmacytoid dendritic cells. It signals by binding to the heterodimeric type I IFN receptor composed of the IFNAR1 and IFNAR2 chains.
- **Interleukin 12 (IL-12)**: proinflammatory interleukin that is naturally produced by dendritic cells, macrophages, neutrophils, and human B-lymphoblastoid cells.
- **C-X-C motif chemokine ligand 10 (CxCL10)**: chemoattractant protein belonging to the chemokine family, attracts and activates cells of the immune system, especially T cells, by binding to its specific receptor, called CXCR3.
- **Interleukin 15 (IL-15)**: proinflammatory cytokine that shares some functional similarities with interleukin-2 and shares its receptor IL-2/IL-15 beta (CD122).
- **Interleukin IL-4 (IL-4)**: antiinflammatory cytokine produced by multiple cell types, such as CD4+ Th2 cells, CD4+NK1.1+ natural T (NKT) cells, macrophages, eosinophils, basophils, mast cells and type 2 innate lymphoid cells (ILC2s); that induces differentiation of naive helper T cells (Th0 cells) to Th2 cells.
- **Interferon gamma (IFN-γ)**: proinflammatory cytokine produced by CD4+ T lymphocytes and natural killer (NK) lymphocytes; critical for innate and adaptive immunity against viral, some bacterial and protozoan infections.
- **Interleukin-1β (IL-1β)**: proinflammatory cytokine produced by activated macrophages, monocytes, and a subset of dendritic cells.
- **Ki67**: nuclear protein that is expressed during various stages in the cell cycle, particularly during late G1, S, G2, and M phases that usually is used like a biomarker of cell proliferation.
- **Knockout (KO):** genetically modified organism, typically a mouse in laboratory research, in which a specific gene has been intentionally deactivated or "knocked out”.
- **Long-term potentiation (LTP)**: is a neurobiological process involving persistent strengthening of synapses that leads to a long-lasting increase in electric signal transmission between neurons described by Bliss and Lomo.
- **Morris water maze (MWM)**: rodent behavioral test designed to assess spatial memory developed by neuroscientist Richard G. Morris.
- **Myelin basic protein (MBP)**: protein that plays an important role in the process of myelination of nerves in the nervous system allowing the electrical insulation to greatly increase the velocity of axonal impulse conduction.
- **Myelin oligodendrocyte glycoprotein (MOG)**: glycoprotein important in the myelination of nerves in the central nervous system
- **Natural killer cells (NK)**: a subtype of lymphocyte cytotoxic of the innate immune system, that do not require prior exposure to specific antigens to become activated.
- **Nestin**: a Class VI intermediate filament abundantly produced in the developing central nervous system (CNS) and in early embryonic neuroepithelial stem cells works like a biomarker for stem/progenitor cells, glioma cells, and tumor endothelial cells.
- **NeuN**: is a 350 amino acid protein also known as Fox-3 that regulates alternative splicing events, it is localized to both the nucleus and cytoplasm, is a biomarker of mature neurons.
- **Neural stem cells (NSCs)**: somatic stem cells in a multipotency state that give rise to neurons, astrocytes, and oligodendrocytes in brain neurogenic niches (subventricular zone and dentate gyrus).
- **Neural progenitor cells (NPCs)**: stem cells in unipotent restricted state that give rise to neurons in brain neurogenic niches (subventricular one and dentate gyrus).
- **Novel Object Recognition Test (NOR)**: rodent behavioral test designed to assess innate curiosity, short-term memory, or long-term memory.
- **Olfactory Epithelium (OE)**: specialized epithelial tissue inside the nasal cavity that allows the smell by detection of odorants through olfactory sensory neurons.
- **Olfactory Bulb (OB)**: neuroanatomical structure located in the forebrain of vertebrates that receives neural input about odors detected by cells in the nasal cavity.
- **Pathogen-associated molecular pattern molecules (PAMPs)**: molecular structures commonly found in bacteria, viruses, fungi, and parasites. These are recognized by the innate immune system as foreign and potentially harmful, triggering an immune response.
- **Proliferating Cell Nuclear Antigen (PCNA)**: essential protein for DNA replication and is involved in DNA excision and mismatch repair pathways, it is a biomarker of early S phase of the cell cycle.
- **Polysialylated-neural cell adhesion molecule (PSA-NCAM)**: is a long homopolymer of sialic acid that is expressed in neural precursors that are migrating and during the process of axonal sprouting, guidance, and targeting, been a marker of developing and migrating neurons and of synaptogenesis in the vertebrate nervous system.
- **Rostral migratory stream (RMS)**: is a pathway in the brain that plays a crucial role in the migration of neuroblast from the subventricular zone to the olfactory bulb in the adult brain.
- **Sox2**: is an intronless gene involved in the regulation of embryonic development and in the determination of cell fate, biomarker of stem-cell in the central nervous system,
- **Subgranular Zone (SGZ)**: is a brain region in the hippocampus located between the granule cell layer and hilus of the dentate gyrus, characterized by abundant neural stem cells.
- **Subventricular Zone (SVZ)**: is a major germinal zone which persists in the adult brain situated on the outside wall of each lateral ventricle of the vertebrate brain abundant on neural stem cells.
- **Cornu Ammonis (CA)**: nomenclature used to subdivide the hippocampus regions, principally composed by glutamatergic pyramidal cells, a key structure in the brain involved in learning and memory.
- **Tumor necrosis factor (TNF-α)**: pro-inflammatory cytokine mainly produced by macrophages and T cells.
- **Interleukin 6 (IL-6)**: regulatory cytokine produced in response to infections and tissue injuries, contributes to host defense through the stimulation of acute phase responses, hematopoiesis, and immune reactions.
- **Neuron-Specific Class III Beta-Tubulin (Tuj1)**: proteína que contribuye a la estabilidad de los microtúbulos en los cuerpos celulares neuronales y los axones, y desempeña un papel en el transporte axonal; está presente de manera dominante en las neuronas tempranas (inmaduras), y funciona como un biomarcador de la diferenciación neuronal más temprana.
- **Vimentin**: also known as fibroblast intermediate filament, is a structural developmentally regulated intermediate filament protein found in cells of mesenchymal origin, involved with the intracellular transport of proteins between the nucleus and plasma membrane.
- **Beta-3 Tubulin (βIIITubulin)**: protein isoform presents dominantly in cells of neuronal origin, and it is one of the earliest markers of neuronal differentiation and mature neurons.
- **Radial Glial-Like cells (RGL)**: pluripotential cells situated in the subgranular zone of the dentate gyrus, with the potential to give rise to astrocytes and neurons.
- **Cerebrospinal Fluid (CSF)**: is a clear, colorless liquid that surrounds the brain and spinal cord, produced in the brain's ventricles by structures called the choroid plexuses.
- **Neurotrophin-3 (NT-3)**: a protein growth factor which is expressed by enteric neural progenitors; it promotes survival of progenitors and differentiation of enteric neurons and glia.
- **Tyrosine Kinase**: an enzyme mediator of signal transduction process, leading to cell proliferation, differentiation, migration, metabolism and programmed cell death.
- **PI3K/Akt pathway**: intracellular pathway important in regulating the cell cycle directly related to cellular quiescence, proliferation, cancer, and longevity.
- **Nuclear factor kappa-light-chain-enhancer of activated B cells (NFkB)**: is a family of transcription factor protein complexes that controls transcription of DNA, cytokine production and cell survival.
- **Fractalkine (CX3CL1)**: is a large cytokine protein also commonly known under the names fractalkine (in humans) and neurotactin (in mice) expressed by some monocytes, it maintains microglia quiescent under physiological conditions, and plays an important roll on neural plasticity and immune response.
- **Fractalkine Receptor (CX3CR1)**: is the receptor that binds to fractalkine (CX3CL1)
- **Intra-cerebro-ventricular (i.c.v.)**: is a route of administration for drugs via injection into the cerebral ventricles so that it reaches the cerebrospinal fluid
- **Colony-stimulating factor 1 receptor antagonist (PLX5622)**: is a potent inhibitor of the kinase activity used to eliminate microglia in the brain.
- **Lymphocyte antigen 6 (Ly6C)**: is an antigen commonly used to identify monocytes/macrophages.
- **PNS:** it is a network of nerves that derive from the CNS that extend to the rest of the body's organs to receive and transmit information, allowing us to respond to stimuli.
- **Nerve growth factor (NGF) :** it is a protein that belongs to the neurotrophin family and promotes the differentiation, growth, and survival of neurons.
- **Glial cell-derived neurotrophic factors (GDNF):** it is part of a family of proteins that bears the same name and is related to neuronal maintenance and development.
- **GFRα1:** is a GDNF receptor, activates the RET receptor protein kinase, thereby initiating several signal transduction pathways.
- **CREB:** it is a transcription factor that stimulates the transcription of genes involved in proliferation, differentiation, and survival.
